# General *N*-and *O*-Linked Glycosylation of Lipoproteins in Mycoplasmas and Role of Exogenous Oligosaccharide

**DOI:** 10.1371/journal.pone.0143362

**Published:** 2015-11-23

**Authors:** James M. Daubenspeck, David S. Jordan, Warren Simmons, Matthew B. Renfrow, Kevin Dybvig

**Affiliations:** 1 Department of Genetics, University of Alabama at Birmingham, Birmingham, Alabama, United States of America; 2 Department of Microbiology, University of Alabama at Birmingham, Birmingham, Alabama, United States of America; 3 Department of Biochemistry and Molecular Genetics, University of Alabama at Birmingham, Birmingham, Alabama, United States of America; Miami University, UNITED STATES

## Abstract

The lack of a cell wall, flagella, fimbria, and other extracellular appendages and the possession of only a single membrane render the mycoplasmas structurally simplistic and ideal model organisms for the study of glycoconjugates. Most species have genomes of about 800 kb and code for few proteins predicted to have a role in glycobiology. The murine pathogens *Mycoplasma arthritidis* and *Mycoplasma pulmonis* have only a single gene annotated as coding for a glycosyltransferase but synthesize glycolipid, polysaccharide and glycoproteins. Previously, it was shown that *M*. *arthritidis* glycosylated surface lipoproteins through *O*-linkage. In the current study, *O*-linked glycoproteins were similarly found in *M*. *pulmonis* and both species of mycoplasma were found to also possess *N*-linked glycans at residues of asparagine and glutamine. Protein glycosylation occurred at numerous sites on surface-exposed lipoproteins with no apparent amino acid sequence specificity. The lipoproteins of *Mycoplasma pneumoniae* also are glycosylated. Glycosylation was dependent on the glycosidic linkages from host oligosaccharides. As far as we are aware, *N*-linked glycoproteins have not been previously described in Gram-positive bacteria, the organisms to which the mycoplasmas are phylogenetically related. The findings indicate that the mycoplasma cell surface is heavily glycosylated with implications for the modulation of mycoplasma-host interactions.

## Introduction

Glycoproteins are ubiquitous in eukaryotes with the number of glycosylated proteins thought to represent over half of the proteome [1]. Once thought of as rare in prokaryotes, protein glycosylation has been shown in numerous bacterial species. The glycans can serve various roles including affects on protein folding and stability, ligand binding, signaling, and cell-cell interactions [2,3]. *O*-linked glycosylation at serine and threonine residues has been found in many species of both Gram-negative and Gram-positive bacteria [2–9]. *N*-linked glycosylation at asparagine residues has been described for many Gram-negative but not Gram-positive bacteria [7,8].

Mycoplasmas are valuable models for the study of membrane biology and the glycosylation of membrane proteins because of the lack of a cell wall and the possession of only a single lipid bilayer. Most species have less than 1,000 genes with few annotated as having a likely role in glycobiology. *Mycoplasma pulmonis* and *Mycoplasma arthritidis* are thought to lack the ability to synthesize nucleotide sugars such as UDP-glucose, but they nevertheless synthesize a variety of glycomoieties [10–12]. The single annotated glycosyltransferase gene identified in these species has not been disrupted in extensive transposon libraries and hence is thought to be essential [13–15]. *O*-linked glycosylation at serine and threonine residues of lipoproteins of *M*. *arthritidis* has been previously reported [11]. The identified glycans were always a single molecule of hexose, either glucose or mannose.

We report here that the addition of disaccharides to the growth medium increased the abundance of glucose in macromolecules of *M*. *pulmonis* and the human pathogen *Mycoplasma pneumoniae*. Glycostaining and GC/MS indicated that supplementation with disaccharides increased the overall level of protein glycosylation. The lipoproteins of *M*. *pulmonis* were examined by high-resolution mass spectrometry (HR-MS) and found to have both *O*-linked and *N*-linked glycosylation. An unusual aspect of the findings is that not just asparagine residues, but also glutamine residues of surface-exposed proteins are glycosylated at numerous sites. The *N*- and *O*-linked glycosites had no apparent sequence specificity. In all reported cases the glycans were a hexose. Retrospective analysis of the lipoproteins of *M*. *arthritidis* resulted in similar findings of *N*-linked glycosylation in addition to the previously reported *O*-linked glycosylation.

## Materials and Methods

### Strains and culture conditions

*M*. *arthritidis* strain TnCtrl, derived from strain 158, was used here and for previous studies on protein glycosylation [11,15,16]. It was cultured in serum-free medium [12]. *M*. *pulmonis* strain CTG [10] and *M*. *pneumoniae* strain M129 [17] were grown in DMEM (Cellgro) supplemented with 15% whole horse serum (Atlantic Biologicals), 0.2% Brain Heart Infusion Powder (Fisher), 0.5% IsoVitaleX (Fisher), 0.02% degraded free-acid DNA (Sigma), and 0.01% ampicillin. Where noted, the medium was supplemented with 0.3% glucose, lactose or maltose.

### Staining of SDS-PAGE gels

Bacteria harvested from cultures in stationary phase were washed twice with phosphate-buffered saline (PBS). Lipoproteins were fractionated using Triton X-114 (TX-114) as previously described [18,19]. Extracted lipoproteins were suspended in 20 μl SDS loading buffer and heated to 100° C. The proteins were electrophoresed on 4–15% gradient SDS-PAGE gels (Bio-Rad). Glycoproteins were stained with the Pro-Q Emerald 300 Glycoprotein Gel and Blot Stain Kit (Invitrogen) following the manufacturer’s protocol. Parallel gels were analyzed for glycoproteins or total protein stained with Coomassie blue.

### GC/MS

Whole cell lysates were digested with nucleases, dialyzed, and analyzed by GC/MS as described [10]. For analysis of lipoprotein fractions, TX-114 was removed from the samples with Pierce® Detergent Removal Spin Columns.

### HR-MS

Protein bands were excised and subjected to in-gel tryptic digestion. The samples were subjected to LC-HR-MS using the instrumentation and methods as described [11]. The mass spectra were analyzed by using the PEAKS 7 proteomics software (Bioinformatics Solutions, Inc.). Each MS peak reported here was obtained from an obvious LC peak that spanned 4 to 5 scans. The PEAKS software manual suggests that peptide scores (-10lgP) of 20 and above are acceptable candidates for manual analysis. In this study, only spectra with scores of 50 or higher were analyzed. In the algorithm searches, asparagine, glutamine, serine, and threonine residues were searched for the presence of hexose (162.0528 Da) in monomeric, dimeric and trimeric forms. A decoy dataset consisting of 8 non-lipoproteins was similarly analyzed. These proteins were MYPU_3210 (oligopeptidase F), MYPU_4280 (elongation factor G), MYPU_5110 (transketolase), MYPU_5180 (enolase), MYPU_7300 (aminopeptidase), MYPU_7610 (dihydrolipoamide dehydrogenase), MYPU_7620 (dihydrolipoamide acetyltransferase), and MYPU_7640 (pyruvate dehydrogenase E1 component). Using our search parameters and PEAKS peptide score criteria, no hexosylation was detected in this decoy data set.

## Results

### Disaccharides support glycoconjugate synthesis

Using ^13^C-labeled glucose and starch, it was previously shown that oligosaccharides but not monosaccharides support the synthesis of glycoconjugates in *M*. *arthritidis*, *M*. *pneumoniae*, *M*. *pulmonis* [11,12]. No ^13^C-labeled glycoconjugates were detected when these species of mycoplasma were grown in medium supplemented with ^13^C monosaccharide glucose, but robust labeling was found when the medium was supplemented with hydrolyzed ^13^C starch. Investigated here was the ability of the disaccharides maltose (α-D-glucopyranosyl-(1→4)-D-glucose) and lactose (β-D-galactopyranosyl-(1→4)-D-glucose) to support glycoconjugate synthesis in *M*. *pulmonis* and *M*. *pneumoniae*.

**Fig 1:**
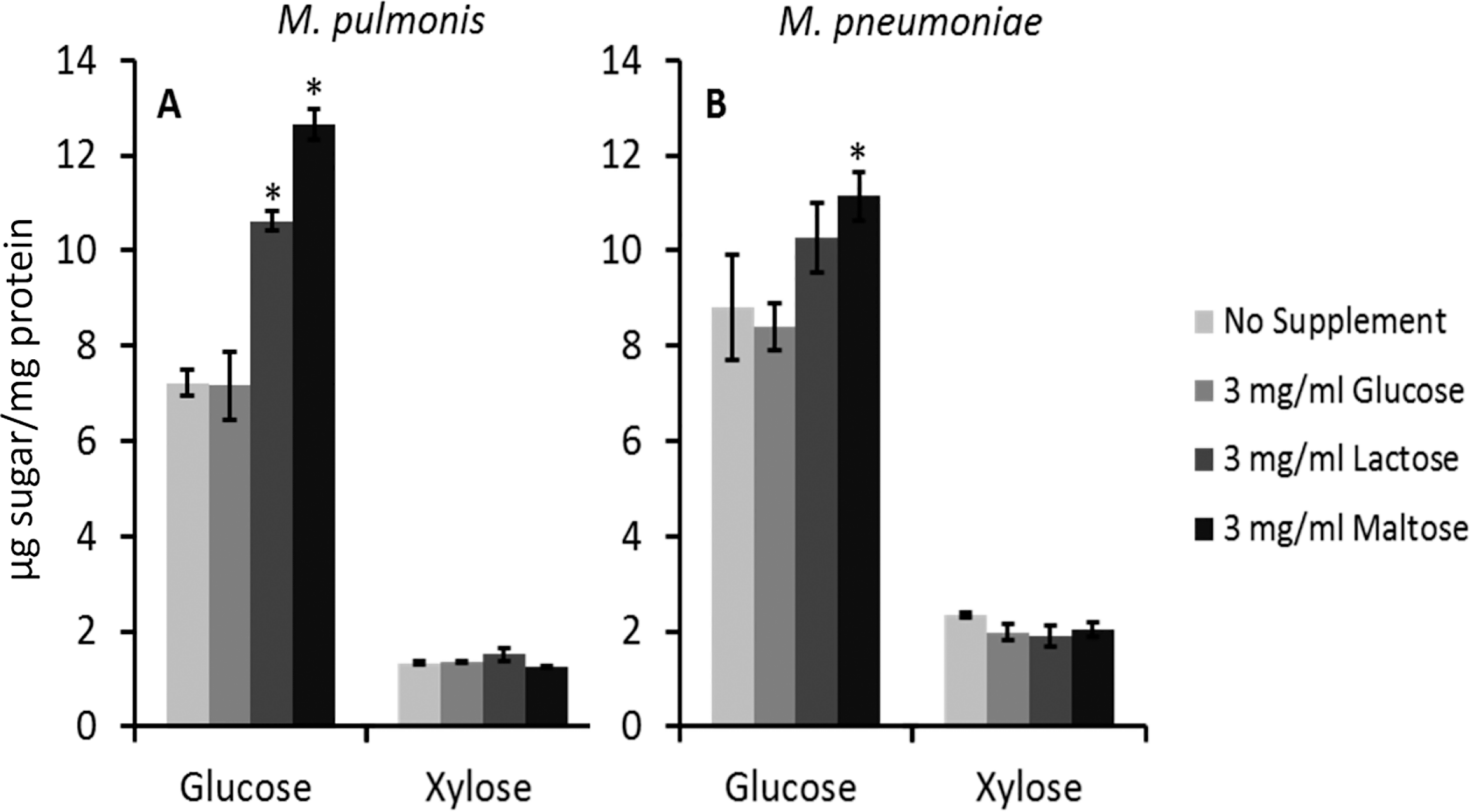
The effect of substrate on glycoconjugate synthesis by *M*. *pulmonis* and *M*. *pneumoniae*. Panels A and B show the relative abundance of glucose or xylose, respectively, linked to protein as determined by GC. The values represent averages of the areas under the curves from 3 replicates of gas chromatograms that were converted to μg of sugar per mg of protein. The colors of the bars representing the different substrates used to supplement the medium are shown on the right. Plus or minus standard error bars are shown and the asterisks indicate a significant difference between a sample and control.

Lysates of mycoplasmas were digested with nucleases and extensively dialyzed to remove monosaccharides and other small molecules. The monosaccharide composition of the glycoconjugates remaining in the lysates were analyzed by GC/MS and compared to standards. The relative abundance of glucose associated with glycoconjugates significantly increased for both *M*. *pulmonis* and *M*. *pneumoniae* when the culture media was supplemented with maltose (*P* < 0.01 and *P* < 0.05, respectively) (Fig 1). Supplementation with lactose also resulted in a significant increase in the abundance of glucose in *M*. *pulmonis* glycoconjugates (*P* < 0.01). To control for the possibility that supplementation with disaccharides affected glycoconjugate synthesis indirectly as a result of enhanced energy metabolism because of an overabundance of glucose, the glycoconjugates were analyzed after growth of the mycoplasmas in medium supplemented with the monosaccharide glucose. Supplementation with glucose had no affect on the relative abundance of the sugars incorporated into glycoconjugates. Xylose was also present in all lysates but its abundance was unaffected by supplementation with glucose, lactose, or maltose. These results demonstrate that the mycoplasmas can utilize both alpha- and beta-linked disaccharides for glycoconjugate synthesis.

### Glycostaining of proteins

**Fig 2:**
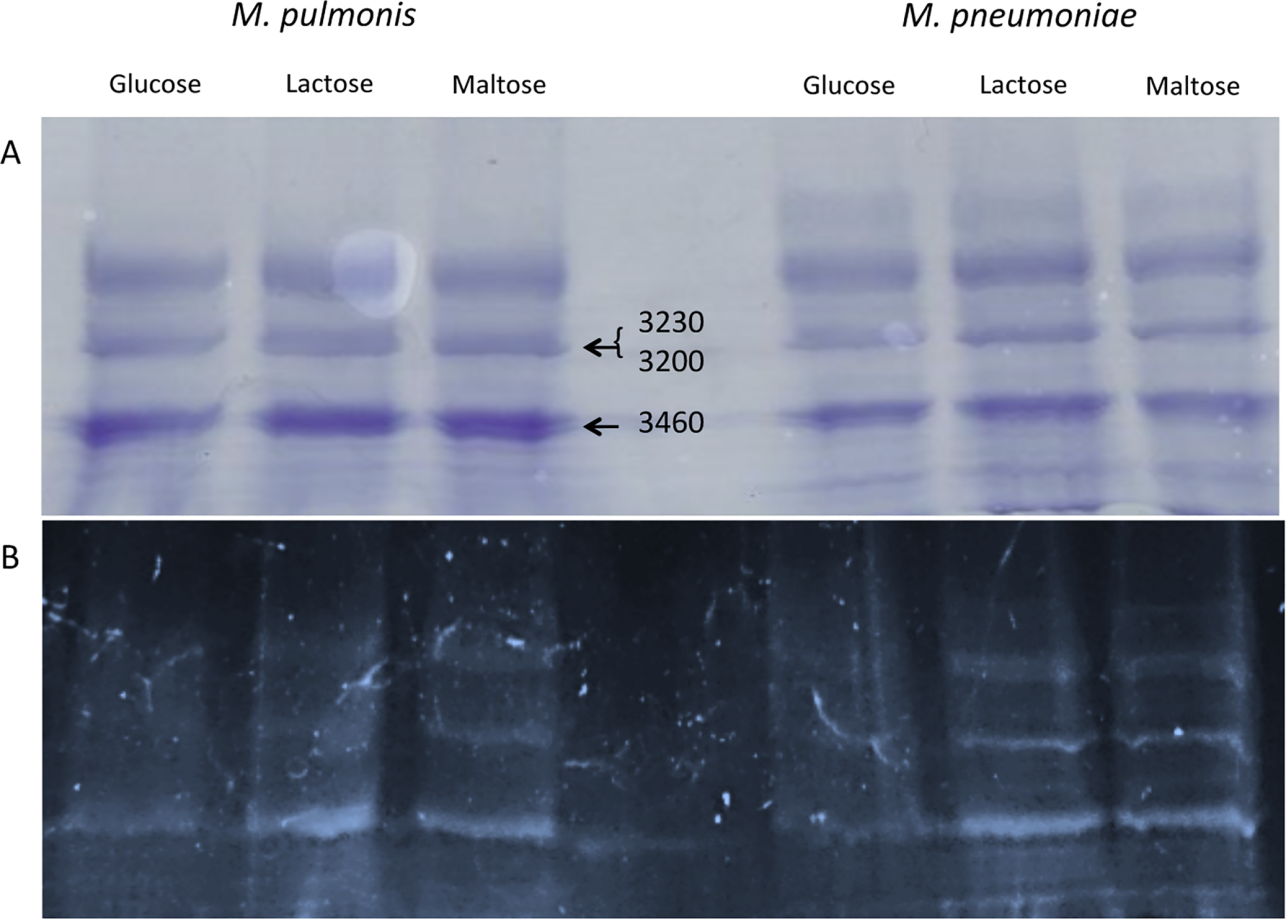
SDS PAGE of TX-114 fractions of *M*. *pulmonis and M*. *pneumoniae*. Panel B shows a glycostained gel, and panel A is a Coomassie-stained gel run in parallel. *M*. *pulmonis* bands excised for further analysis by HR-MS are indicated by arrows with the designation of the genes coding for the identified proteins. The unlabeled top band was analyzed by HR-MS and while there were several spectra that suggested glycosylation, none met our criteria for publication.

Proteins of *M*. *pulmonis* and *M*. *pneumoniae* were extracted with TX-114 to fractionate lipoproteins from non-lipoproteins. Proteins from the detergent phase, containing lipoproteins, were electrophoresed on SDS-PAGE gels that were stained with Pro-Q Emerald 300 to identify potential glycoproteins or Coomassie blue to identify total proteins. The banding pattern of the lipoprotein fraction was highly similar for the two stains (Fig 2A and 2B), suggesting that most of the lipoproteins were glycosylated as previously found for *M*. *arthritidis* [11]. Glycostaining was increased for proteins from cultures supplemented with maltose or lactose, suggesting again that glycosynthesis in mycoplasmas is dependent on oligosaccharides.

Two doublets of proteins of *M*. *pulmonis* grown in medium supplemented with maltose were excised from the Coomassie-stained gels and analyzed by HR-MS. The major proteins were found to be MYPU_3200, MYPU_3230, and MYPU_3460. The MS coverage for these major proteins was in excess of 90%. Each of these proteins contains a signal peptide sequence indicative of a lipoprotein (see GenBank accession number NC_002771 for the predicted amino acid sequences, also available at http://services.cbib.u-bordeaux2.fr/molligen/).

### Interpretation of HR-MS spectra

**Fig 3:**
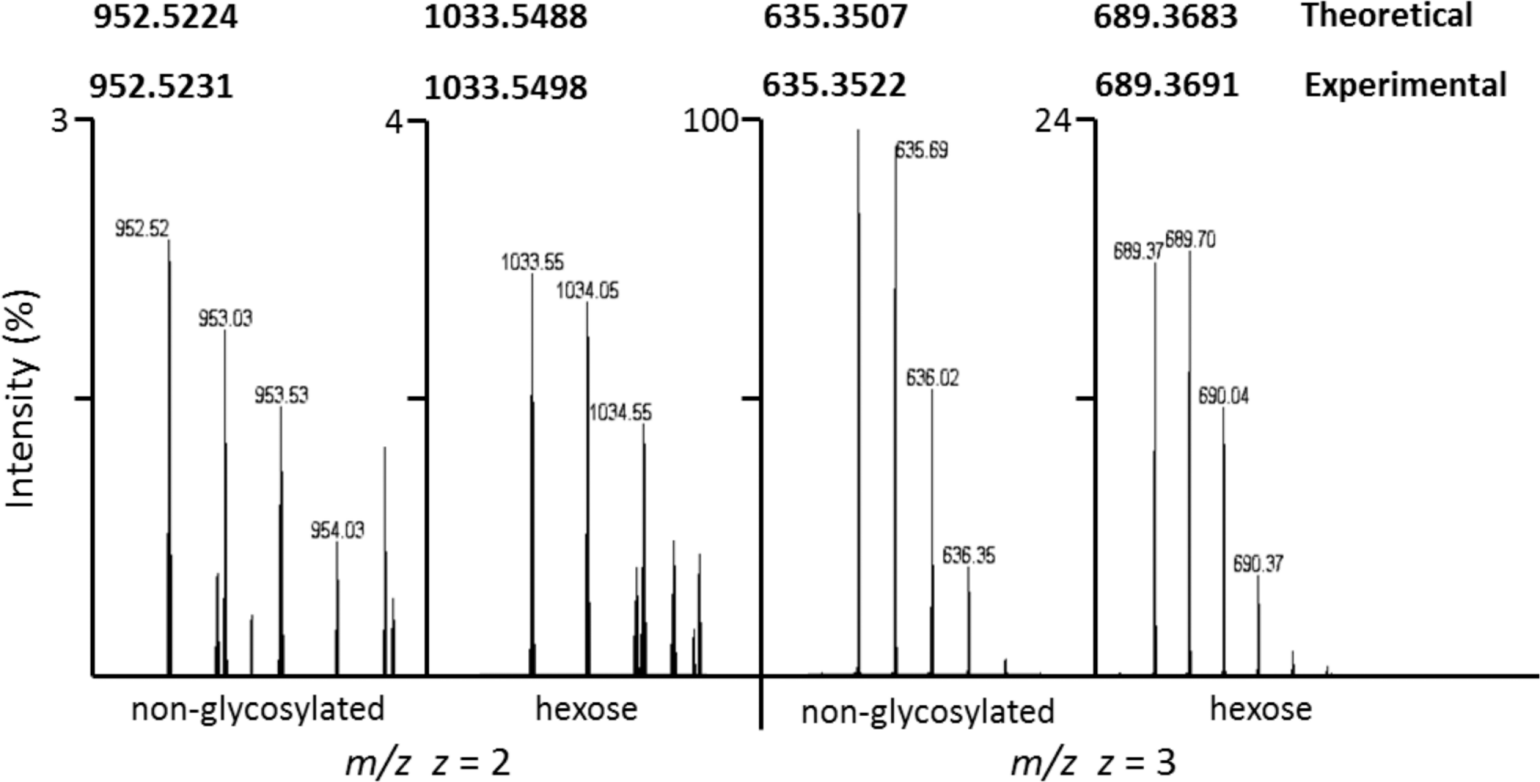
Hexosylation of the peptide GTKDFLPIELQSLEVSK of MYPU_3230. Orbitrap MS showing the doubly and triply charged ions. The 81.0262 shift for *z* = 2 between the non-glycosylated and glycosylated peptides equates to a mass shift of 162.0524 Da, which corresponds to the addition of hexose (162.0528 Da) with a mass accuracy of 0.0004 Da. The 54.0169 shift for *z* = 3 between non-glycosylated and glycosylated forms equates to a mass shift of 162.0507 Da, which corresponds to hexosylation with a mass accuracy of 0.0021 Da. Monoisotopic values for the calculated theoretical and experimental masses of the peptide are given in bold. The images presented were obtained from an LC peak of MS scans and are expanded to show the charge states of each form.

The mass of the centroid for each HR-MS peak was calculated as described by Gedcke [20]. As an example, an expanded view of the peak at 952.52 Da in the left panel of Fig 3 is shown in S1 Fig. The digitized data were stored in 8 bins with the abundance of the events in a bin and the mass of the bin as indicated in the middle and right panels of the figure. The data are stored essentially as a histogram with the mass of the centroid calculated from the equation show in S1 Fig. Assuming a Gaussian distribution, the calculated mass at the centroid has a standard deviation equal to the full width of the peak at half maximum height divided by 2.35. The height and width of the peak are uncertain because of the information lost when the data were digitized, but we estimate the standard deviation to be about 0.008 Da for each of the spectra presented in this paper. The estimated mass as calculated from the centroid of the MS peak for all of the peptides agrees with the expected mass calculated from the amino acid sequence to a level of accuracy much smaller than the standard deviation.

### *O*-linked glycosylation of *M*. *pulmonis* proteins

**Fig 4:**
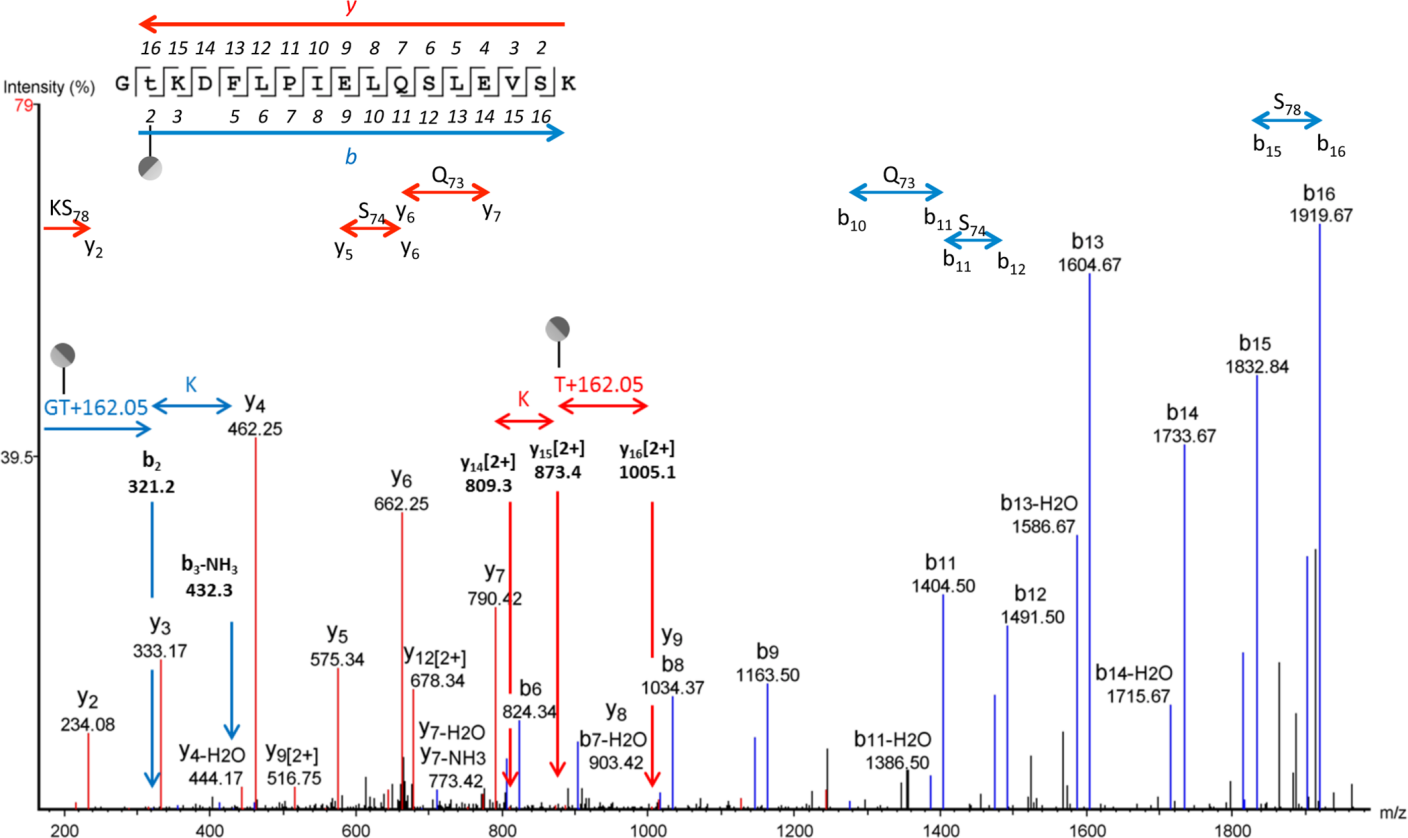
LC MS/MS-CID showing hexosylation at Thr64 of the peptide Gt_64_KDFLPIELQSLEVSK of MYPU_3230. The assigned b and y ions are shown in blue and red, respectively. Glycosylation of Q and S glycosites is absent in this spectrum as illustrated. The PEAKS peptide score (-10lgP) for this spectrum was 87. The charge state of the parental ion was *z* = 3.

Previously, we identified *O*-linked hexosylation at serine and threonine residues of the lipoproteins MARTH_455, MARTH_403, MARTH_665, and MARTH_819 of *M*. *arthritidis* [11]. In the current study, the lipoproteins identified above from *M*. *pulmonis* were examined by HR-MS and fragmented by collision-induced dissociation (CID). Multiple sites of hexose addition at serine and threonine were found for each lipoprotein (see Table 1). The expected mass shift corresponding to addition of a single residue of hexose is 162.0528 Da, which is the mass of the monosaccharide (180 Da) minus the mass of the water molecule that is removed by formation of the glycosidic bond. The HR-MS spectra for the peptide GTKDFLPIELQSLEVSK containing Thr64 of MYPU_3230 is shown in Fig 3. We identified both the hexosylated and non-glycosylated forms in multiple charge states (*z* = 2 and *z* = 3), showing the glycosylated form has a mass shift consistent with the addition of hexose with an accuracy of 0.0004 Da for *z* = 2 and an accuracy of 0.0021 Da for *z* = 3. The relevant calculations are shown in S2 Fig. As a point of reference, the mass of an electron is about 0.0005 Da. A liquid chromatography (LC) MS/MS-CID spectrum showing hexosylation at Thr64 of MYPU_3230 is shown in Fig 4. The mass for the assigned b_2_ ion is 162 Da greater than would be expected for the non-glycosylated form. Similarly, the mass of the assigned y_15_ ion has a mass shift of 162. In both cases, the mass shift occurs at Thr64. The assigned peaks for the spectrum are given in S1 Table.

**Table 1:** Glycosites identified in this study.

Protein^a^	Peptide sequence	MS/MS	MS
MYPU_3200	STLEYTINNSQELQn_335_ILKQTYEEFTK	Fig 6, S2 Table	Fig 5
	AKADLEs_515_LISSK	S4 Fig	S6 Fig
	ADLESLISs_519_KEK	S5 Fig	S7 Fig
MYPU_3230	Gt_64_KDFLPIELQSLEVSK	Fig 4, S1 Table	Fig 3
	TDTAMq_331_ELLKNTYEEFTK	S14 Fig	S15 Fig
MYPU_3460	ITDLLSq_49_KEVTETQK	Fig 8, S4 Table	Fig 7
MARTH_403	ANAKn_495_FYGFSDAYGK	S11 Fig, S3 Table	S10 Fig
	LELAKq_113_VILTLDDGTVK	S17 Fig, S5 Table	S16 Fig

^a^ Accession numbers for MYPU_3200, MYPU_3230, MYPU_3460, and MARTH_403 are CAC13493, CAC13496, CAC13519, and YP_001999972, respectively.

To further validate the results, the MS/MS-CID spectrum of the glycosylated peptide shown in Fig 4 was compared to that of the non-glycosylated peptide (see S3 Fig). The highly similar fragmentation patterns confirm that the spectra are from the same peptide sequence. The y_6_ and b_6_ ions have essentially the same mass in the non-glycosylated spectrum but differ in the glycosylated spectrum by a 162-Da. shift in b_6_. All of the b ions greater than b_6_ were shifted by 162 Da, indicating hexosylation at Thr64.

The *O*-linked glycosylation sites identified in MYPU_3200 were Ser515 and Ser519. The MS/MS spectra for these sites are shown in S4 and S5 Figs. The MS scans are shown in S6 and S7 Figs.

### *N*-glycosylation at asparagine in *M*. *pulmonis* and *M*. *arthritidis*

**Fig 5:**
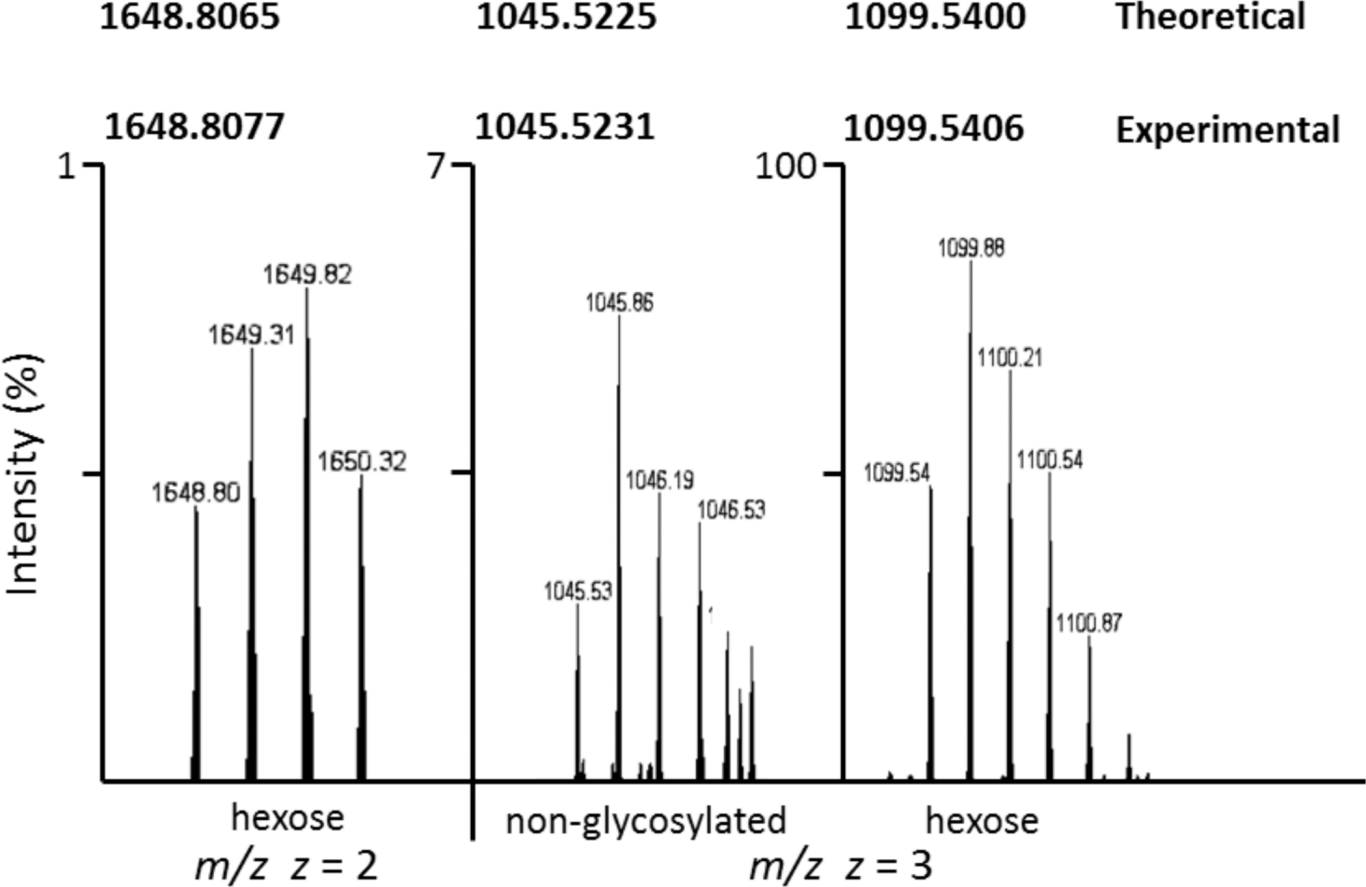
Hexosylation of the peptide STLEYTINNSQELQNILKQTYEEFTK of MYPU_3200. Orbitrap MS showing the doubly and triply charged ions. The monoisotopic mass of the doubly charged species at 1648.8077 is consistent with the hexosylated peptide at *z* = 2 with a mass accuracy of 0.0012 Da. The 54.0175 shift for *z* = 3 between non-glycosylated and hexose forms equates to a mass shift of 162.0525 Da with a mass accuracy of 0.0003 Da. Monoisotopic values for the calculated theoretical and experimental masses of the peptide are given in bold. The images presented were obtained from an LC peak of MS scans and are expanded to show the charge states of each form.

**Fig 6:**
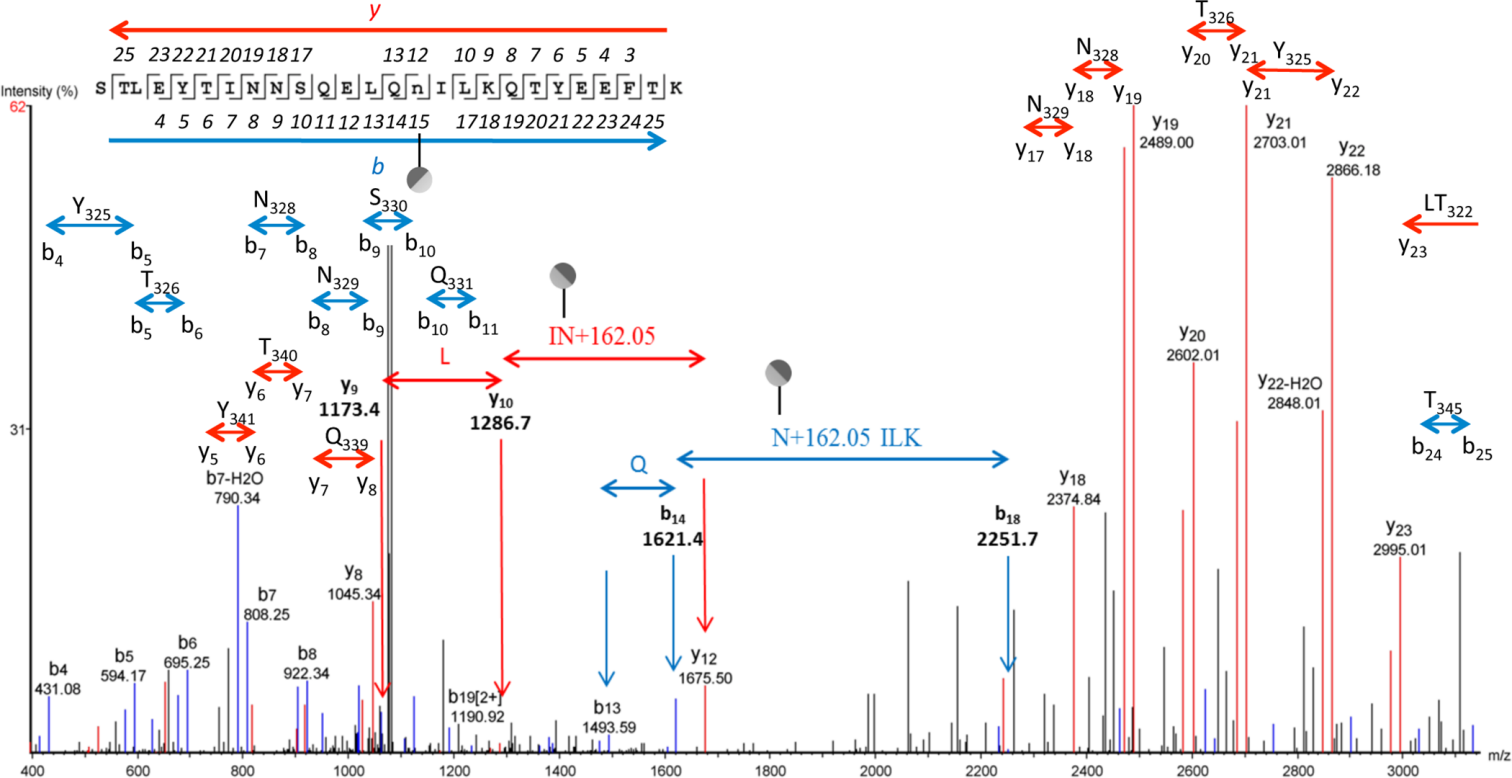
LC MS/MS-CID showing hexosylation at Asn335 of the peptide STLEYTINNSQELQn_335_ILKQTYEEFTK of MYPU_3200. The assigned b and y ions are shown in blue and red, respectively. Glycosylation of N, Q, T, S, and Y glycosites is absent in this spectrum as illustrated. The PEAKS peptide score (-10lgP) for this spectrum was 60. The charge state of the parental ion was *z* = 3.

In addition to *O*-glycosylation, strong evidence for *N*-linked hexosylation was found for the lipoproteins analyzed from *M*. *pulmonis*. The HR-MS spectrum for the peptide containing Asn335 from MYPU_3200 is shown in Fig 5. We identified both the hexosylated and non-glycosylated forms showing the glycosylated form has a mass shift consistent with the addition of hexose with an accuracy of 0.0004 Da for *z* = 3 and the hexosylated form *z* = 2 for the peptide STLEYTINNSQELQNILKQTYEEFTK. A MS/MS spectrum showing *N*-glycosylation at Asn335 of MYPU_3200 is shown in Fig 6. The b_13_ and b_14_ ions clearly delineate the Gln334 residue. The b_18_ ion to b_13_ ion separation gives a clear view of the N+162ILK portion of the sequence. The y_9_ and y_10_ ions as assigned delineate the Leu337 residue. The y_12_ ion shows a mass shift consistent with hexosylation at Asn335. Ion peaks for amino acid residues that are unutilized potential glycosylation sites are highlighted in Fig 6. The list of assigned peaks for the MS/MS spectra is given in S2 Table.

The MS/MS-CID spectrum for the non-glycosylated peptide STLEYTINNSQELQNILK is shown in panel A of S8 Fig, which is a tryptic truncation of the peptide shown in Fig 6 and S8 Fig, panel B. As discussed below and reported by others [21], a single hexose modification in proximity to lysine is sufficient to block trypsin cleavage. Perhaps because of the internal lysine, no spectra were identified for the full-length, non-glycosylated peptide STLEYTINNSQELQNILKQTYEEFTK. For S8 Fig, nonapplicable y ions and extraneous peaks were removed for clarity. The unaltered spectra of these peptides are available in Fig 6 and S9 Fig. The similarity in the fragmentation pattern for the spectra shown in S8 Fig is striking, as expected for peptides that share the same amino acid sequence.

We previously reported widespread *O*-glycosylation of lipoproteins of *M*. *arthritidis* [11] but not *N*-glycosylation. Using recently acquired PEAKS mass spectrometry software (Bioinformatics Solutions Inc., Waterloo, ON), mass spectra for the MARTH_403 lipoprotein were reanalyzed to search for *N*-glycosylation. As for *M*. *pulmonis*, numerous examples of hexosylation at asparagine were found. The HR-MS spectrum for the peptide ANAKNFYGFSDAYGK containing Asn495 from MARTH_403 is shown in S10 Fig. A LC MS/MS-CID spectrum showing glycosylation at Asn495 of MARTH_403 is shown in S11 Fig, and a list of the assigned peaks for this spectrum is given in S3 Table.

### *N*-glycosylation at glutamine in *M*. *pulmonis* and *M*. *arthritidis*

**Fig 7:**
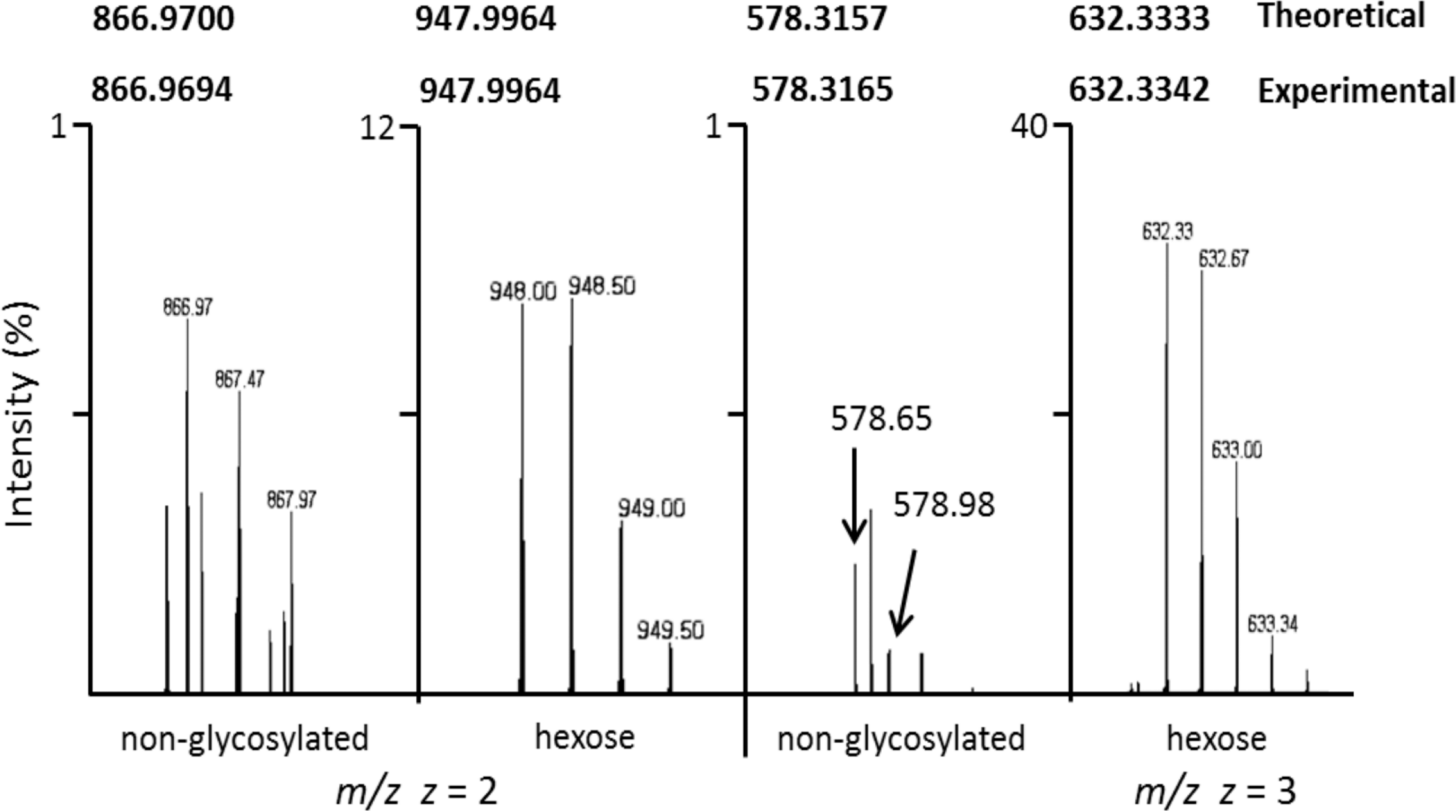
Hexosylation of the peptide ITDLLSQKEVTETQK of MYPU_3460. Orbitrap MS showing the doubly and triply charged ions. The 81.027 shift for *z* = 2 between the non-glycosylated and glycosylated peptides equates to a mass shift of 162.054 Da, which corresponds to the addition of hexose (162.0528 Da) with a mass accuracy of 0.0012 Da. The 54.0177 shift for *z* = 3 between non-glycosylated and glycosylated forms equates to a mass shift of 162.0531 Da, which corresponds to hexosylation with a mass accuracy of 0.0003 Da. The calculated theoretical and experimental values for *m/z* are given in bold. The images presented were obtained from an LC peak of MS scans and are expanded to show the charge states of each form.

**Fig 8:**
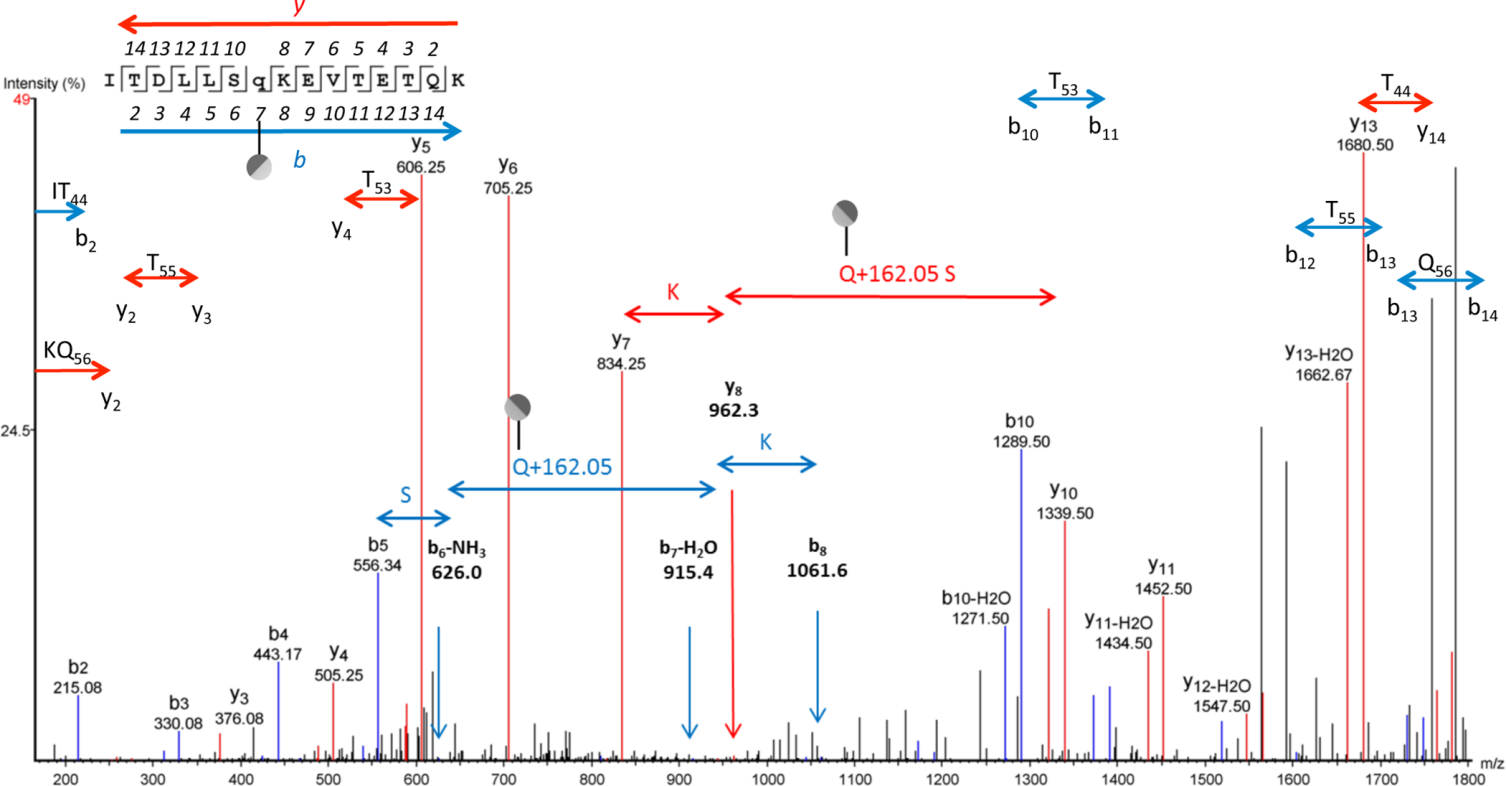
LC MS/MS-CID showing hexosylation at Gln49 of the peptide ITDLLSq_49_KEVTETQK of MYPU_3460. The assigned b and y ions are shown in blue and red, respectively. Glycosylation of Q, S, and T glycosites is absent in this spectrum as illustrated. The PEAKS peptide score (-10lgP) for this spectrum was 74. The charge state of the parental ion was *z* = 3.

Although only rare instances of *N*-linkage to glutamine have been reported for any organism [22,23], examples of hexosylation at glutamine were found in both *M*. *pulmonis* and *M*. *arthritidis*. The HR-MS spectrum for the peptide ITDLLSQKEVTETQK is shown in Fig 7. We identified both the hexosylated and non-glycosylated forms showing the glycosylated form has a mass shift consistent with the addition of hexose with an accuracy of 0.0012 Da for *z* = 2 and the addition of a hexose with an accuracy of 0.0003 Da for *z* = 2. A spectrum showing glycosylation at Gln49 for the peptide ITDLLSQKEVTETQK of MYPU_3460 is shown in Fig 8. The assigned peaks for b_5_ (556.3), b_6_-NH3 (626.0), b_7_-H_2_O (915.4), and b_8_ (1061.6) provide a clear view of the residues Ser48, Gln49+162.05, and Lys50. The assigned peaks at y_7_ (834.3), y_8_ (962.3) and y_10_ (1339.5) provide delineation in the other direction. The assigned peaks for this spectrum are shown in S4 Table.

The MS/MS-CID spectrum for the non-glycosylated peptide ITDLLSQK is shown in panel A of S12 Fig, which is a tryptic truncation of the peptide shown in Fig 8. The glycosylated peptide ITDLLSQKEVTETQK showing hexosylation at Gln49 is shown in panel B for comparison. As for S8 Fig, nonapplicable y ions and extraneous peaks have been removed for clarity and the unaltered spectra are available in Fig 8 and S13 Fig. The similarities between the spectra are obvious. The major difference between the spectra is the increase in the b_2_ ion in panel A and the shift at the b_7_ ion by 162 Da., indicative of glycosylation at Gln49.

Hexosylation was also identified at Gln331 of the *M*. *pulmonis* protein MYPU_3230. The MS/MS spectrum is shown in S14 Fig, and the MS scans are provided in S15 Fig.

Another example of hexosylation at glutamine was identified on reanalysis of MARTH_403 data. The MS spectrum for the peptide LELAKQVILTLDDGTVK is shown in S16 Fig and the MS/MS showing glycosylation at Gln113 is shown in S17 Fig. The assigned peaks for the MS/MS spectrum are shown in S5 Table.

## Discussion

Our findings show that *M*. *arthritidis* and *M*. *pulmonis* possess both general *N*- and *O*-protein glycosylation. The similar findings obtained for these two species suggest that general protein glycosylation may be widespread in mycoplasmas. As far as we are aware, the mycoplasmas are the first group of Gram-positive bacteria for which *N*-glycosylation has been described. The lack of amino acid sequence specificity at the glycosites of the mycoplasmal proteins is surprising. *O*-glycosylation in mycoplasmas lacked the three amino acid motif D(S/T)(A/I/L/M/T/V) found in *Bacteroides* [24], and *N*-glycosylation in mycoplasmas lacked the NX(ST) sequon found in most organisms [8,25]. Glutamine glycosylation appears to be commonplace in mycoplasmas but is considered rare in other organisms [22]. With glycosylation at asparagine, glutamine, serine and threonine residues and no obvious amino acid sequence specificity at the glycosites, the mycoplasmas have the most general protein glycosylation reported for any organism.

No peptide was identified as having two glycosylated amino acids on the same molecule. Perhaps one hexosylation event shields the adjacent amino acids from further glycosylation. The proposed system would generate a high level of diversity with not all molecules glycosylated at the same sites and may represent a new type of antigenic variation that contributes to the chronicity of infection.

In addition to the possibility that a glycosylation event inhibits further glycosylation at neighboring glycosites, glycosylation might also inhibit cleavage by proteases at nearby sites. All of the glycopeptides listed in Table 1 have internal lysine residues that in theory could have been cleaved by the trypsin reaction that generated the glycopeptides. These lysine residues are frequently near the site of glycosylation. Similarly, most of the previously reported glycosites found in proteins of *M*. *arthritidis* were in the vicinity of a lysine residue [11]. Perhaps the positively-charged amino acid is required for enzymatic recognition. In general, HR-MS analysis of the smaller peptides that were generated by cleavage at these internal residues failed to identify glycosylation events. It is probable that proteins that were non-glycosylated were cleaved by trypsin more efficiently than the glycosylated proteins because it has been shown previously that glycosylation of proteins with single residues of glucose inhibits cleavage by trypsin [21]. Mycoplasmas are scavengers that do not synthesize amino acids *de novo* and acquire them from the host through the import of oligopeptides that are generated by the activity of proteases secreted by the mycoplasma [26,27]. The general protein glycosylation system described here could have a critical role in protecting the mycoplasmal cell surface from these proteases as has been proposed for other bacterial glycosylation systems [28].

The glycosites reported here represent a small subset of the sites for which we have strong evidence for glycosylation of the lipoproteins of *M*. *arthritidis* and *M*. *pulmonis*. Dozens of additional glycosites could be identified by further analysis of the mass spectra. The sites reported here are limited to the abundant lipoproteins that were examined and those sites that were glycosylated at sufficient frequency to yield a high number of MS/MS scans that clearly showed the modification. Potential minor sites for glycosylation were ignored in the current study.

Oligosaccharides, not monosaccharides, support glycoconjugate synthesis in *M*. *pulmonis* and *M*. *arthritidis* [12]. Mycoplasmas are dependent on a host to provide many essential nutrients such as cholesterol, amino acids, and nucleotides. The only source of oligosaccharides available to the mycoplasma would also be the animal host. In this study we found that oligosaccharides containing alpha- and beta- 1,4 linkages supported protein glycosylation in *M*. *pulmonis* and *M*. *pneumoniae*. The full range of oligosaccharide substrates that could be used by the mycoplasmas may be broad because of the complexity of the glycoconjugates encountered in the host. Current thinking is that the energy stored in the glycosidic bond of the oligosaccharides drives glycoconjugate synthesis, abrogating the need of nucleotide sugars. The oligosaccharides supplied in the culture medium or by the animal host are used to build the glycoconjugates that the mycoplasma synthesizes. The glycocalyx of the mycoplasmal cell surface might resemble closely that of the host milieu, perhaps contributing to immune avoidance. This simple strategy of decorating surface proteins with glycans obtained from host oligosaccharides is economical, as the mycoplasmas do not need to synthesize the glycans themselves or expend energy to produce nucleotide sugar substrates. This survival strategy is utilized in the human intestinal commensal, *Bacteroides fragilis*, which decorates surface-exposed proteins and polysaccharides with host-derived fucose [29].

Lactose is not imported by *M*. *pulmonis* [13] and yet its addition to the medium increased the level of protein glycosylation. Furthermore, there is no evidence for hexosylation of cytoplasmic proteins in mycoplasmas and hexosylation occurs at many sites in a fashion that appears to be stochastic. Hence, we propose that glycosylation of the lipoproteins occurs extracellularly. Extracellular glycosylation in mycoplasmas using host oligosaccharides as substrates would resemble oligosaccharide synthesis in some oral bacteria that use sucrose and glucans as substrates [30]. The enzymes that are responsible for synthesis of these glycans are glycosidases/glycosyltransferases that transfer the sugar residue from an oligosaccharide to the bacterial substrate. In the *M*. *pulmonis* proteome there are five predicted glycosidases (MYPU_1030, 4630, 6330, 6340, 6440) that could potentially glycosylate the lipoproteins. As the membranes of *M*. *pulmonis* cells are often in intimate contact with host cell membrane [31], the possibility exists that not just mycoplasmal proteins but also host proteins are targets of the glycosyltransferase(s) produced by mycoplasmas.

## Supporting Information

S1 Fig HR-MS of the peptide GTKDFLPIELQSLEVSK of MYPU_3230. Panel A is the *m/z* = 2 for this peptide. Panel B is an expanded view of the 952.52 peak from panel A displaying the mass and relative abundance of each bin in which the digitized data are stored. Panel C illustrates the histogram of the data from which the centroid mass is calculated as shown in the equation as described by Gedke, 2001.
(PDF)

S2 Fig Calculations for hexosylation of Thr_64_ in the peptide GTKDFLPIELQSLEVSK of MYPU_3230. Monoistopic mass for peptide was calculated utilizing ExPASy PeptideMass (web.expasy.org/peptide_mass/). Monoisotopic mass for monosaccharides was calculated utilizing ACD/ChemSketch Software.
(PDF)

S3 Fig LC MS/MS-CID showing the non-glycosylated (A) and the hexosylated (B) forms of the peptide GTKDFLPIELQSLEVSK. (PDF)

S4 Fig LC MS/MS-CID showing hexosylation at Ser515 of the peptide AKADLEs_515_LISSK of MYPU_3200. The assigned b and y ions are shown in blue and red, respectively. Glycosylation of S glycosites is absent in this spectrum as illustrated. The PEAKS peptide score (-10lgP) for this spectrum was 52. The charge state of the parental ion was *z* = 3.
(PDF)

S5 Fig LC MS/MS-CID showing hexosylation at Ser519 peptide ADLESLISs_519_KEKof MYPU_3200. The assigned b and y ions are shown in blue and red, respectively. Glycosylation of S glycosites is absent in this spectrum as illustrated. The PEAKS peptide score (-10lgP) for this spectrum was 57. The charge state of the parental ion was *z* = 3.
(PDF)

S6 Fig Hexosylation of the peptide AKADLESLISSK of MYPU_3200. Orbitrap MS1 showing the doubly and triply charged ions. The 81.0264 shift for *z* = 2 between the non-glycosylated and hexose peptides equates to a mass shift of 162.0528 Da with a mass accuracy of 0.0008 Da. The 54.0181 shift for *z* = 3 between non-glycosylated and hexose forms equates to a mass shift of 162.0543 Da with a mass accuracy of 0.0015 Da. The theoretical and experimental calculated values for *m/z* are given in bold. The images presented were obtained from an LC peak of MS scans and are expanded to show the charge states of each form.
(PDF)

S7 Fig Hexosylation of the peptide ADLESLISSKEK of MYPU_3200. Orbitrap MS1 showing the doubly and triply charged ions. The 81.0268 shift for *z* = 2 between the non-glycosylated and hexose peptides equates to a mass shift of 162.0536 Da with a mass accuracy of 0.0008 Da. The 54.0170 shift for *z* = 3 between non-glycosylated and hexose forms equates to a mass shift of 162.0510 Da with a mass accuracy of 0.0018 Da. The theoretical and experimental calculated values for *m/z* are given in bold. The images presented were obtained from an LC peak of MS scans and are expanded to show the charge states of each form.
(PDF)

S8 Fig LC MS/MS-CID showing the non-glycosylated truncated (A) and the hexosylated (B) forms of the peptide STLEYTINNSQELQNILK(QTYEEFTK). Nonapplicable y ions and extraneous peaks have been removed for clarity.
(PDF)

S9 Fig LC MS/MS-CID raw data corresponding to S8 Fig panel A. (PDF)

S10 Fig Hexosylation of the peptide ANAKNFYGFSDAYGK of MARTH_403. Orbitrap MS showing the doubly and triply charged ions. The 81.0277 shift for *z* = 2 between the non-glycosylated and glycosylated peptides equates to a mass shift of 162.0554 Da, which corresponds to the addition of a hexose (162.0528 Da) with a mass accuracy of 0.0026 Da. The 54.0181 shift for *z* = 3 between non-glycosylated and glycosylated forms equates to a mass shift of 162.0543 Da, which indicates the addition of a hexose with a mass accuracy of 0.0015 Da. The theoretical and experimental calculated values for *m/z* are given in bold. The images presented were obtained from an LC peak of MS scans and are expanded to show the charge states of each form.
(PDF)

S11 Fig LC MS/MS-CID showing hexosylation at Asn495 of the peptide ANAKn_495_FYGFSDAYGK of MARTH_403. The assigned b and y ions are shown in blue and red, respectively. Glycosylation of N, S, and Y glycosites is absent in this spectrum as illustrated. The PEAKS peptide score (-10lgP) for this spectrum was 66. The charge state of the parental ion was *z* = 3.
(PDF)

S12 Fig LC MS/MS-CID showing the non-glycosylated truncated (A) and the hexosylated (B) forms of the peptide ITDLLSQK(EVTETQK). Nonapplicable y ions and extraneous peaks have been removed for clarity.
(PDF)

S13 Fig LC MS/MS-CID raw data corresponding to S12 Fig panel A. (PDF)

S14 Fig LC MS/MS-CID showing hexosylation at Gln331 of the peptide TDTAMq_331_ELLKNTYEEFTKof MYPU_3230. LC The assigned b and y ions are shown in blue and red, respectively. Glycosylation of T and N glycosites is absent in this spectrum as illustrated. The PEAKS peptide score (-10lgP) for this spectrum was 62. The charge state of the parental ion was z = 3.
(PDF)

S15 Fig Hexosylation of the peptide TDTAMQELLKNTYEEFTK of MYPU_3230. Orbitrap MS1 showing the doubly and triply charged ions. The monoisotopic mass of the doubly charged species at 1162.5453 is consistent with the hexosylated peptide at *z* = 2 with a mass accuracy of 0.0009 Da. The 54.0169 shift for *z* = 3 between non-glycosylated and hexose forms equates to a mass shift of 162.0507 Da with a mass accuracy of 0.0021 Da. The oxidation at M_330_ was detected for the (z = 3) hexosylated peptide for both the sulfoxide (O) and sulfone (O_2_) forms. The theoretical and experimental calculated values for *m/z* are given in bold. The images presented were obtained from an LC peak of MS scans and are expanded to show the charge states of each form.
(PDF)

S16 Fig Hexosylation of the peptide LELAKQVILTLDDGTVK of MARTH_403. Orbitrap MS showing the doubly and triply charged ions. The 81.0277 shift for *z* = 2 between the non-glycosylated and glycosylated peptides equates to a mass shift of 162.0554 Da, which corresponds to the addition of a hexose (162.0528 Da) with a mass accuracy of 0.0026 Da. The 54.0174 shift for *z* = 3 between non-glycosylated and glycosylated forms equates to a mass shift of 162.0522 Da, which is consistent with the addition of a hexose with a mass accuracy of 0.0006 Da. The theoretical and experimental calculated values for *m/z* are given in bold. The images presented were obtained from an LC peak of MS scans and are expanded to show the charge states of each form.
(PDF)

S17 Fig LC MS/MS-CID showing hexosylation at Gln113 of the peptide LELAKq_113_VILTLDDGTVK of MARTH_403. The assigned b and y ions are shown in blue and red, respectively. Glycosylation of T glycosites is absent in this spectrum as illustrated. The PEAKS peptide score (-10lgP) for this spectrum was 66. The charge state of the parental ion was *z* = 3.
(PDF)

S1 Table MS/MS peak assignments for the peptide Gt_64_KDFLPIELQSLEVSK of MYPU_3230. (PDF)

S2 Table MS/MS peak assignments for the peptide STLEYTINNSQELQn_335_ILKQTYEEFTK of MYPU_3200. (PDF)

S3 Table MS/MS peak assignments for the peptide ANAKn_495_FYGFSDAYGK of MARTH_403. (PDF)

S4 Table MS/MS peak assignments for the peptide ITDLLSq_49_KEVTETQK of MYPU_3460. (PDF)

S5 Table MS/MS peak assignments for the peptide LELAKq_113_VILTLDDGTVK of MARTH_40. (PDF)
