# Supplementary material for: General N-and O-Linked Glycosylation of Lipoproteins in Mycoplasmas and Role of Exogenous Oligosaccharide
Source: PLoS One. 2015 Nov 23;10(11):e0143362. doi: 10.1371/journal.pone.0143362 (PMC4657876; doi:10.1371/journal.pone.0143362)
Supplement: S1 Fig — Panel A is the m/z = 2 for this peptide. Panel B is an expanded view of the 952.52 peak from panel A displaying the mass and relative abundance of each bin in which the digitized data are stored. Panel C illustrates the histogram of the data from which the centroid mass is calculated as shown in the equation as described by Gedke, 2001. (PDF) [file pone.0143362.s001.pdf]

S1 Figure

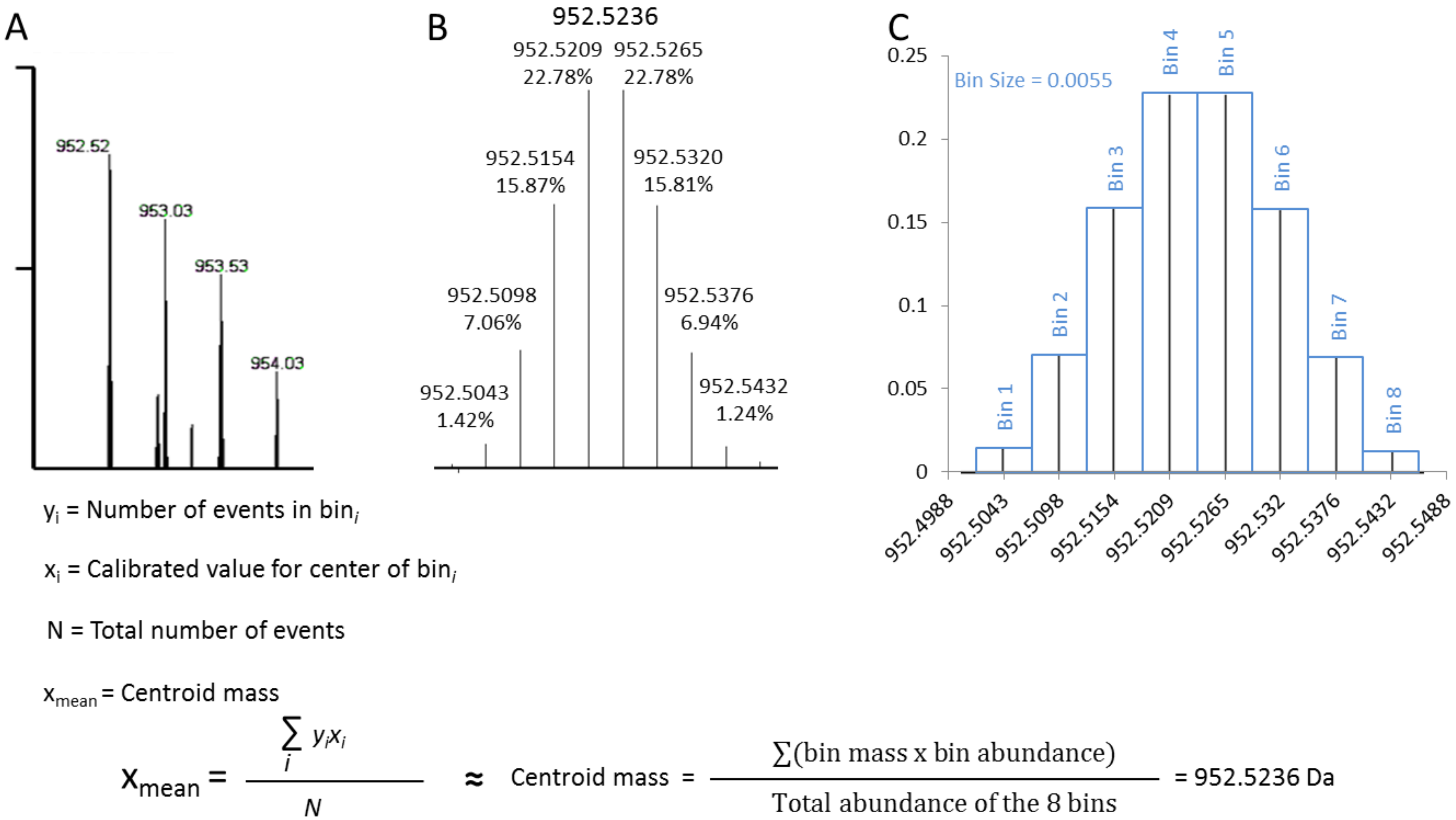

y_i = Number of events in bin$_i$

x_i = Calibrated value for center of bin$_i$

N = Total number of events

x_{mean} = Centroid mass

$$x_{mean} = \frac{\sum_i y_i x_i}{N} \approx \text{Centroid mass} = \frac{\sum(\text{bin mass x bin abundance})}{\text{Total abundance of the 8 bins}} = 952.5236 \text{ Da}$$

S1 Fig. HR-MS of the peptide GTKDFLPIELQSLEVSK of MYPU_3230. Panel A is the *m/z* = 2 for this peptide. Panel B is an expanded view of the 952.52 peak from panel A displaying the mass and relative abundance of each bin in which the digitized data are stored. Panel C illustrates the histogram of the data from which the centroid mass is calculated as shown in the equation as described by Gedke, 2001.
