# Supplementary material for: General N-and O-Linked Glycosylation of Lipoproteins in Mycoplasmas and Role of Exogenous Oligosaccharide
Source: PLoS One. 2015 Nov 23;10(11):e0143362. doi: 10.1371/journal.pone.0143362 (PMC4657876; doi:10.1371/journal.pone.0143362)
Supplement: S2 Fig — Monoistopic mass for peptide was calculated utilizing ExPASy PeptideMass (web.expasy.org/peptide_mass/). Monoisotopic mass for monosaccharides was calculated utilizing ACD/ChemSketch Software. (PDF) [file pone.0143362.s002.pdf]

S2 Figure
Mass Calculations for GTKDFLPIELQSLEVSK

Monoisotopic Mass
GTKDFLPIELQSLEVSK = 1903.0302
Hexose = 162.0528
H = 1.0073

MS1 Formula
(Peptide + Hexose + z*H)/z = Mass

MS1 non-glycosylated z = 2
(1903.0302 + 2 * 1.0073)/2 = 952.5224

MS1 glycosylated z = 2
(1903.0302 + 162.0528 + 2 * 1.0073)/2 = 1033.5488

MS1 non-glycosylated z = 3
(1903.0302 + 3 * 1.0073)/3 = 635.3507

MS1 glycosylated z = 3
(1903.0302 + 162.0528 + 3 * 1.0073)/3 = 689.3683

S2 Fig. Calculations for hexosylation of Thr_{64} in the peptide GTKDFLPIELQSLEVSK of MYPU_3230. Monoistopic mass for peptide was calculated utilizing ExPASy PeptideMass (web.expasy.org/peptide_mass/). Monoisotopic mass for monosaccharides was calculated utilizing ACD/ChemSketch Software.
