# Supplementary material for: General N-and O-Linked Glycosylation of Lipoproteins in Mycoplasmas and Role of Exogenous Oligosaccharide
Source: PLoS One. 2015 Nov 23;10(11):e0143362. doi: 10.1371/journal.pone.0143362 (PMC4657876; doi:10.1371/journal.pone.0143362)
Supplement: S3 Fig — (PDF) [file pone.0143362.s003.pdf]

S3 Figure

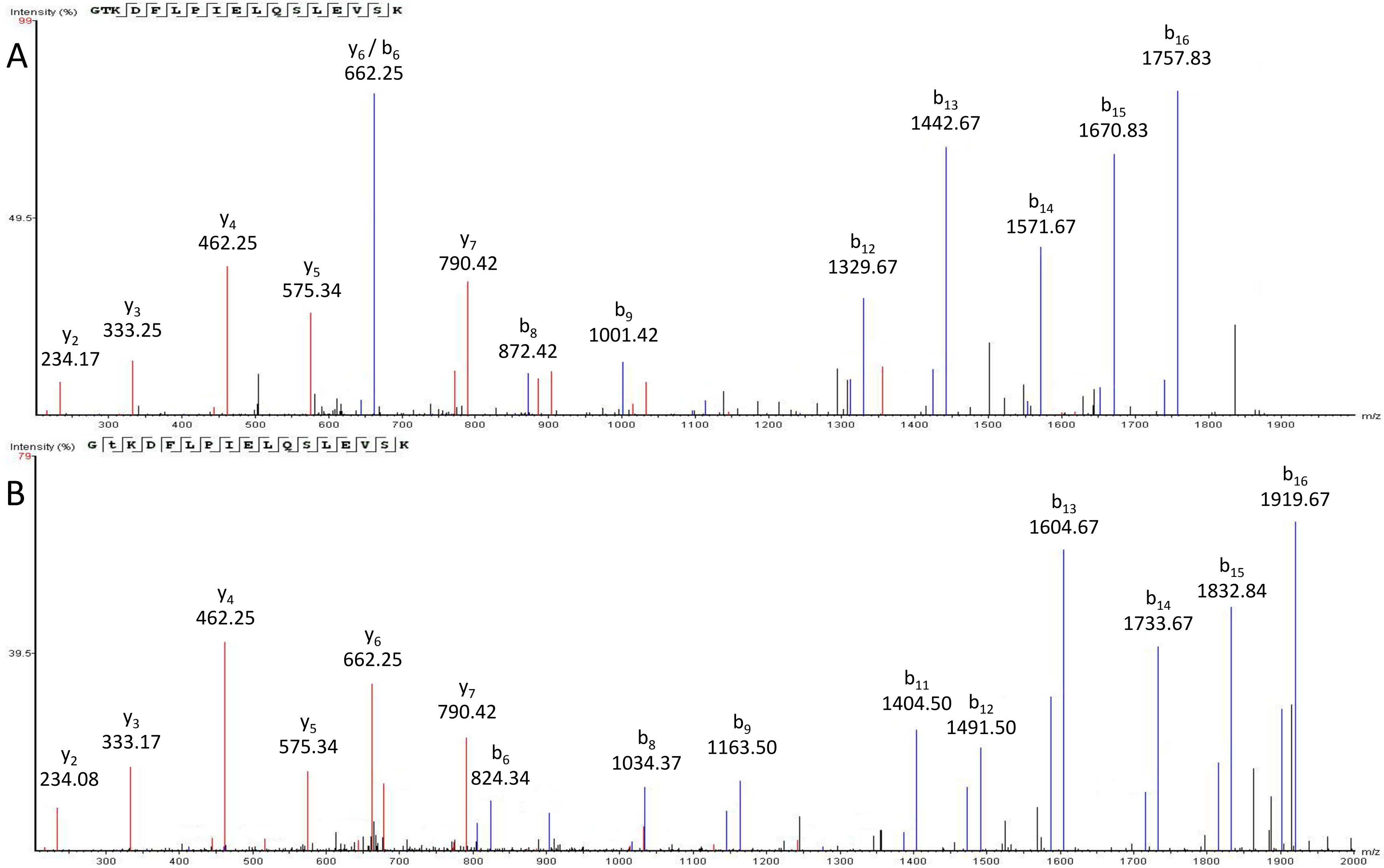

S3 Fig. LC MS/MS-CID showing the non-glycosylated (A) and the hexosylated (B) forms of the peptide GTKDFLPIELQSLEVSK.
