# Supplementary material for: General N-and O-Linked Glycosylation of Lipoproteins in Mycoplasmas and Role of Exogenous Oligosaccharide
Source: PLoS One. 2015 Nov 23;10(11):e0143362. doi: 10.1371/journal.pone.0143362 (PMC4657876; doi:10.1371/journal.pone.0143362)
Supplement: S7 Fig — Orbitrap MS1 showing the doubly and triply charged ions. The 81.0268 shift for z = 2 between the non-glycosylated and hexose peptides equates to a mass shift of 162.0536 Da with a mass accuracy of 0.0008 Da. The 54.0170 shift for z = 3 between non-glycosylated and hexose forms equates to a mass shift of 162.0510 Da with a mass accuracy of 0.0018 Da. The theoretical and experimental calculated values for m/z are given in bold. The images presented were obtained from an LC peak of MS scans and are expanded to show the charge states of each form. (PDF) [file pone.0143362.s007.pdf]

S7 Figure

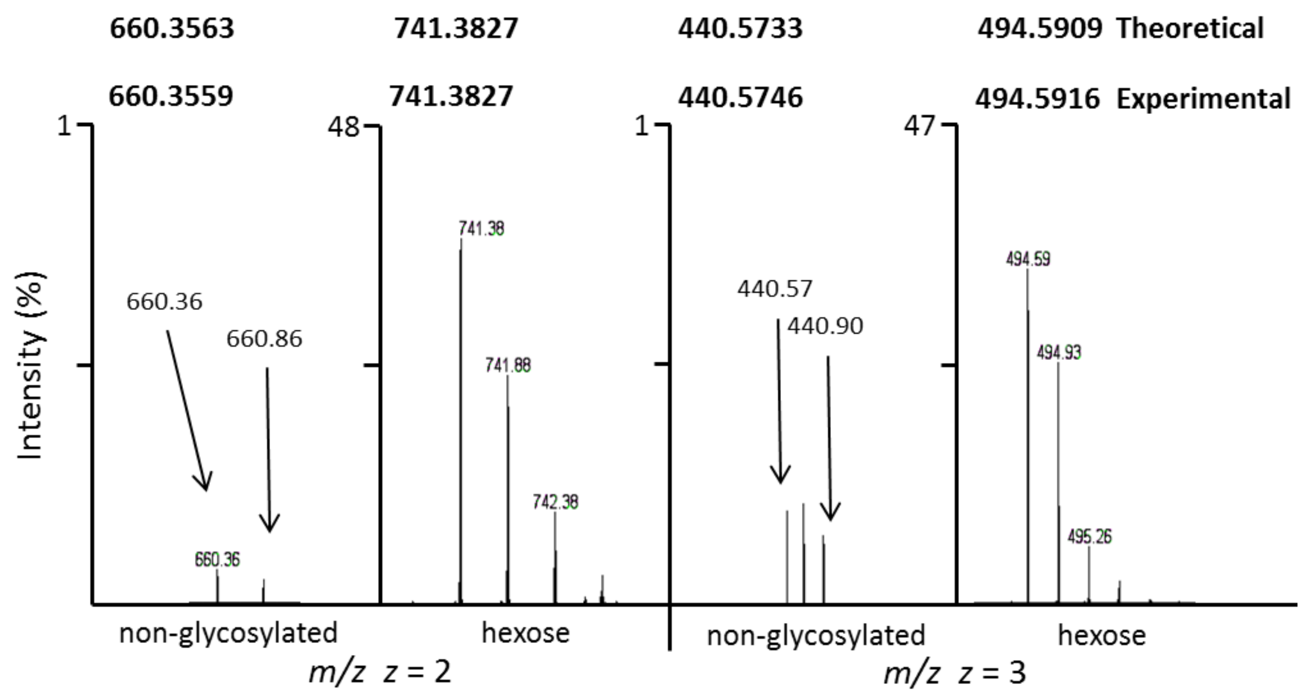

S7 Fig. Hexosylation of the peptide ADLESLISSKEK of MYPU_3200. Orbitrap MS1 showing the doubly and triply charged ions. The 81.0268 shift for $z = 2$ between the non-glycosylated and hexose peptides equates to a mass shift of 162.0536 Da with a mass accuracy of 0.0008 Da. The 54.0170 shift for $z = 3$ between non-glycosylated and hexose forms equates to a mass shift of 162.0510 Da with a mass accuracy of 0.0018 Da. The theoretical and experimental calculated values for *m/z* are given in bold. The images presented were obtained from an LC peak of MS scans and are expanded to show the charge states of each form.
