# Supplementary material for: General N-and O-Linked Glycosylation of Lipoproteins in Mycoplasmas and Role of Exogenous Oligosaccharide
Source: PLoS One. 2015 Nov 23;10(11):e0143362. doi: 10.1371/journal.pone.0143362 (PMC4657876; doi:10.1371/journal.pone.0143362)
Supplement: S8 Fig — Nonapplicable y ions and extraneous peaks have been removed for clarity. (PDF) [file pone.0143362.s008.pdf]

S8 Figure

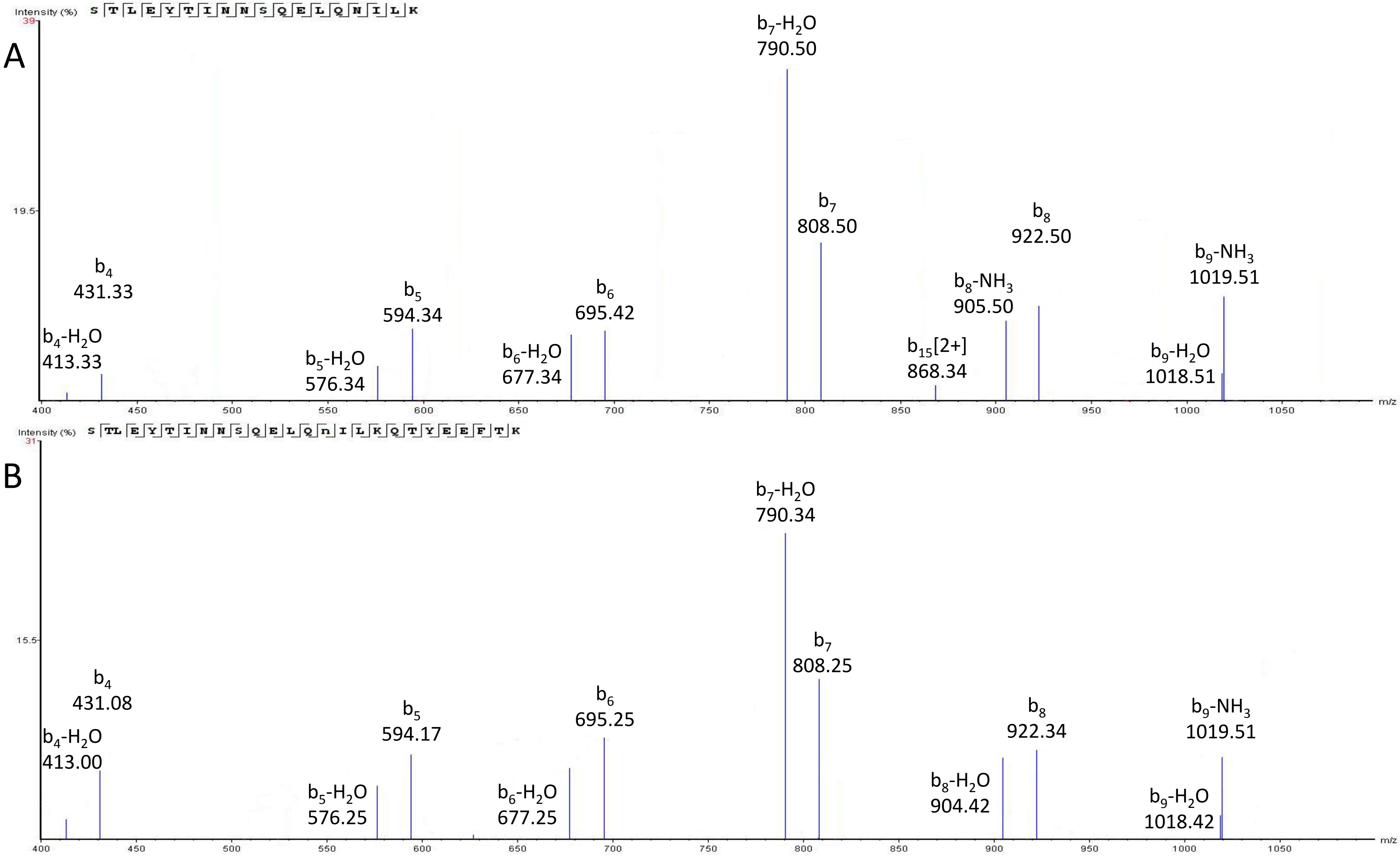

S8 Fig. LC MS/MS-CID showing the non-glycosylated truncated (A) and the hexosylated (B) forms of the peptide STLEYTINNSQELQNILK(QTYEEFTK). Nonapplicable y ions and extraneous peaks have been removed for clarity.
