# Supplementary figures and images for: General N-and O-Linked Glycosylation of Lipoproteins in Mycoplasmas and Role of Exogenous Oligosaccharide
Source: PLoS One. 2015 Nov 23;10(11):e0143362. doi: 10.1371/journal.pone.0143362 (PMC4657876; doi:10.1371/journal.pone.0143362)

## S9 Figure

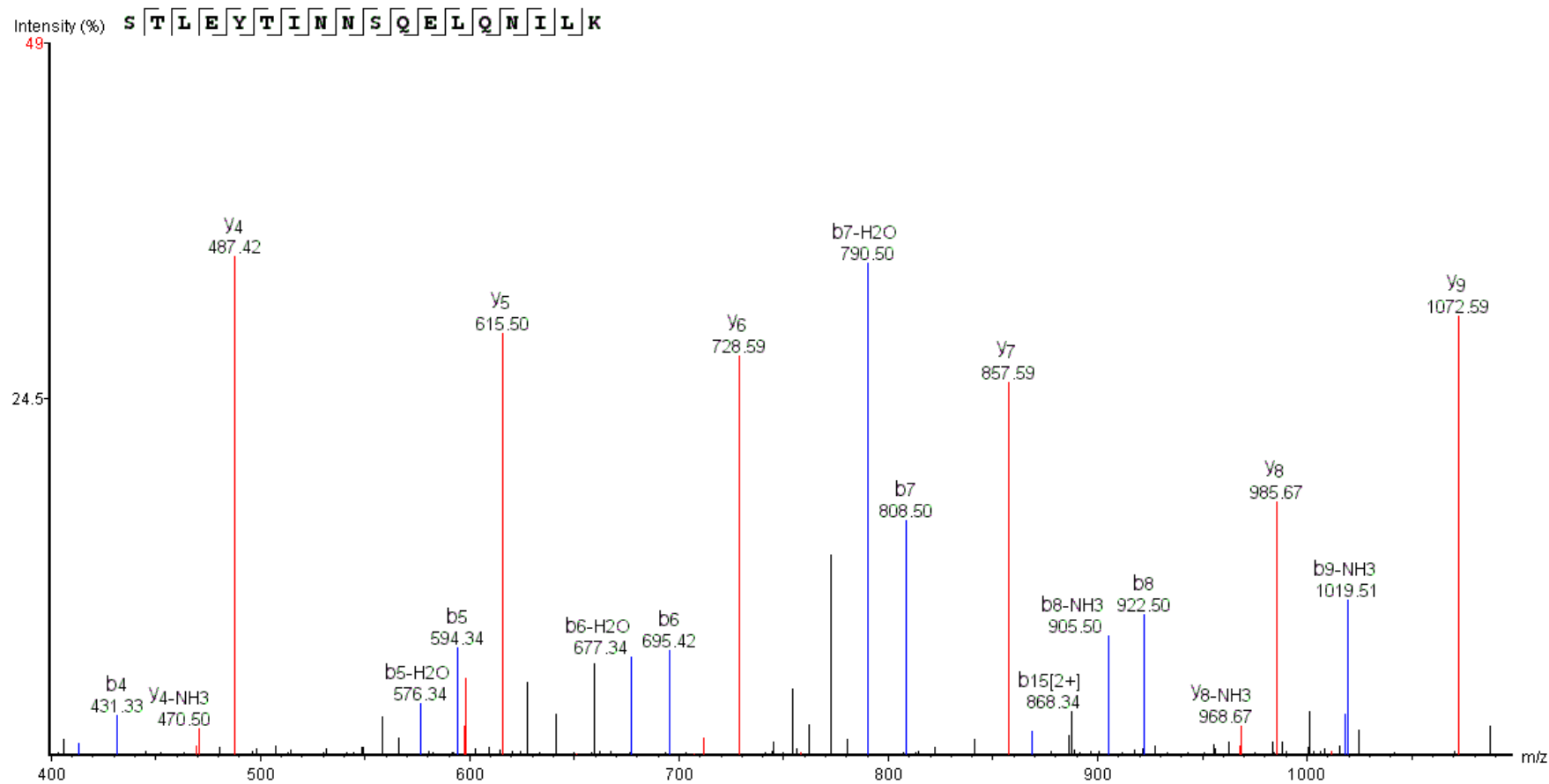

**S9 Fig.** LC MS/MS-CID raw data corresponding to S8 Fig panel A.

Supplement: S9 Fig — (PDF) [file pone.0143362.s009.pdf]

## S13 Figure

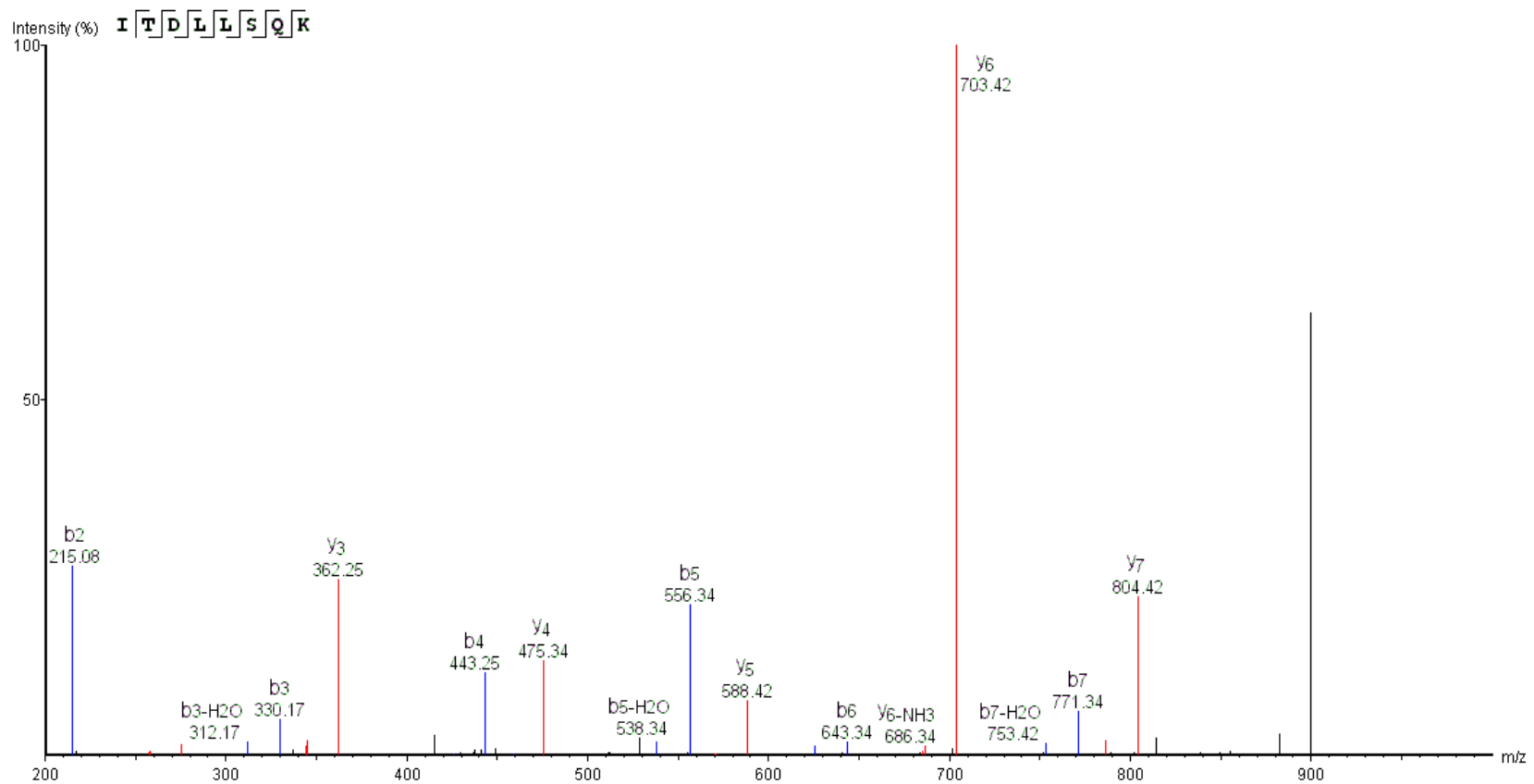

**S13 Fig.** LC MS/MS-CID raw data corresponding to S12 Fig panel A.

Supplement: S13 Fig — (PDF) [file pone.0143362.s013.pdf]
