# Supplementary material for: General N-and O-Linked Glycosylation of Lipoproteins in Mycoplasmas and Role of Exogenous Oligosaccharide
Source: PLoS One. 2015 Nov 23;10(11):e0143362. doi: 10.1371/journal.pone.0143362 (PMC4657876; doi:10.1371/journal.pone.0143362)
Supplement: S15 Fig — Orbitrap MS1 showing the doubly and triply charged ions. The monoisotopic mass of the doubly charged species at 1162.5453 is consistent with the hexosylated peptide at z = 2 with a mass accuracy of 0.0009 Da. The 54.0169 shift for z = 3 between non-glycosylated and hexose forms equates to a mass shift of 162.0507 Da with a mass accuracy of 0.0021 Da. The oxidation at M330 was detected for the (z = 3) hexosylated peptide for both the sulfoxide (O) and sulfone (O2) forms. The theoretical and experimental calculated values for m/z are given in bold. The images presented were obtained from an LC peak of MS scans and are expanded to show the charge states of each form. (PDF) [file pone.0143362.s015.pdf]

S15 Figure

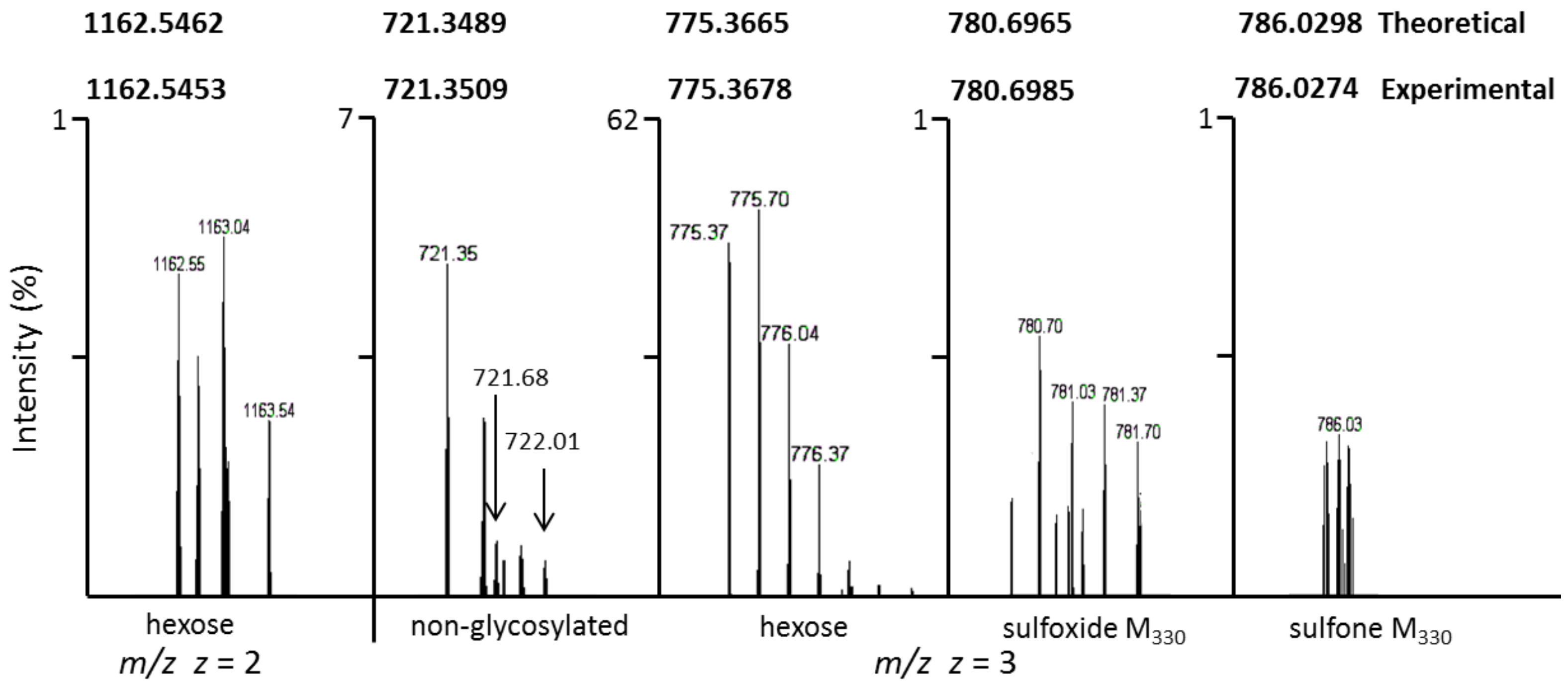

S15 Fig. Hexosylation of the peptide TDTAMQELLKNTYEEFTK of MYPU_3230. Orbitrap MS1 showing the doubly and triply charged ions. The monoisotopic mass of the doubly charged species at 1162.5453 is consistent with the hexosylated peptide at $z = 2$ with a mass accuracy of 0.0009 Da. The 54.0169 shift for $z = 3$ between non-glycosylated and hexose forms equates to a mass shift of 162.0507 Da with a mass accuracy of 0.0021 Da. The oxidation at M_{330} was detected for the (z = 3) hexosylated peptide for both the sulfoxide (O) and sulfone (O_2) forms. The theoretical and experimental calculated values for *m/z* are given in bold. The images presented were obtained from an LC peak of MS scans and are expanded to show the charge states of each form.
