# Supplementary material for: General N-and O-Linked Glycosylation of Lipoproteins in Mycoplasmas and Role of Exogenous Oligosaccharide
Source: PLoS One. 2015 Nov 23;10(11):e0143362. doi: 10.1371/journal.pone.0143362 (PMC4657876; doi:10.1371/journal.pone.0143362)
Supplement: S16 Fig — Orbitrap MS showing the doubly and triply charged ions. The 81.0277 shift for z = 2 between the non-glycosylated and glycosylated peptides equates to a mass shift of 162.0554 Da, which corresponds to the addition of a hexose (162.0528 Da) with a mass accuracy of 0.0026 Da. The 54.0174 shift for z = 3 between non-glycosylated and glycosylated forms equates to a mass shift of 162.0522 Da, which is consistent with the addition of a hexose with a mass accuracy of 0.0006 Da. The theoretical and experimental calculated values for m/z are given in bold. The images presented were obtained from an LC peak of MS scans and are expanded to show the charge states of each form. (PDF) [file pone.0143362.s016.pdf]

S16 Figure

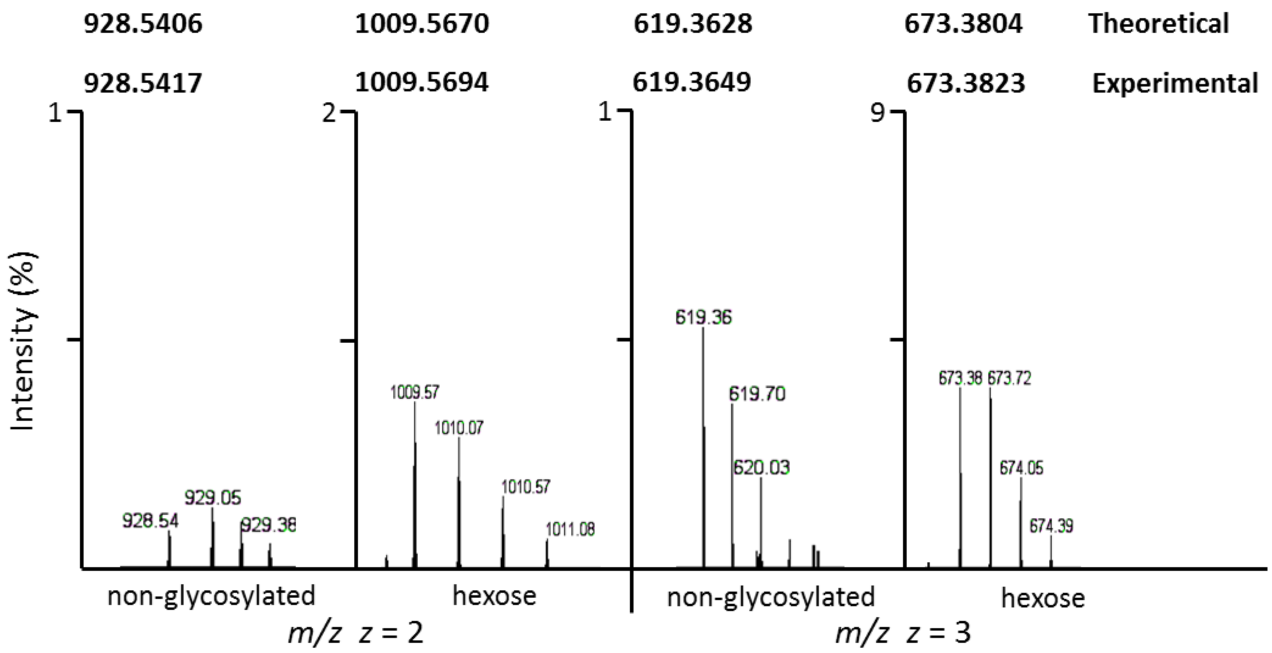

S16 Fig. Hexosylation of the peptide LELAKQVILTLDDGTVK of MARTH_403. Orbitrap MS showing the doubly and triply charged ions. The 81.0277 shift for $z = 2$ between the non-glycosylated and glycosylated peptides equates to a mass shift of 162.0554 Da, which corresponds to the addition of a hexose (162.0528 Da) with a mass accuracy of 0.0026 Da. The 54.0174 shift for $z = 3$ between non-glycosylated and glycosylated forms equates to a mass shift of 162.0522 Da, which is consistent with the addition of a hexose with a mass accuracy of 0.0006 Da. The theoretical and experimental calculated values for *m/z* are given in bold. The images presented were obtained from an LC peak of MS scans and are expanded to show the charge states of each form.
