# Supplementary material for: General N-and O-Linked Glycosylation of Lipoproteins in Mycoplasmas and Role of Exogenous Oligosaccharide
Source: PLoS One. 2015 Nov 23;10(11):e0143362. doi: 10.1371/journal.pone.0143362 (PMC4657876; doi:10.1371/journal.pone.0143362)
Supplement: S17 Fig — The assigned b and y ions are shown in blue and red, respectively. Glycosylation of T glycosites is absent in this spectrum as illustrated. The PEAKS peptide score (-10lgP) for this spectrum was 66. The charge state of the parental ion was z = 3. (PDF) [file pone.0143362.s017.pdf]

S17 Figure

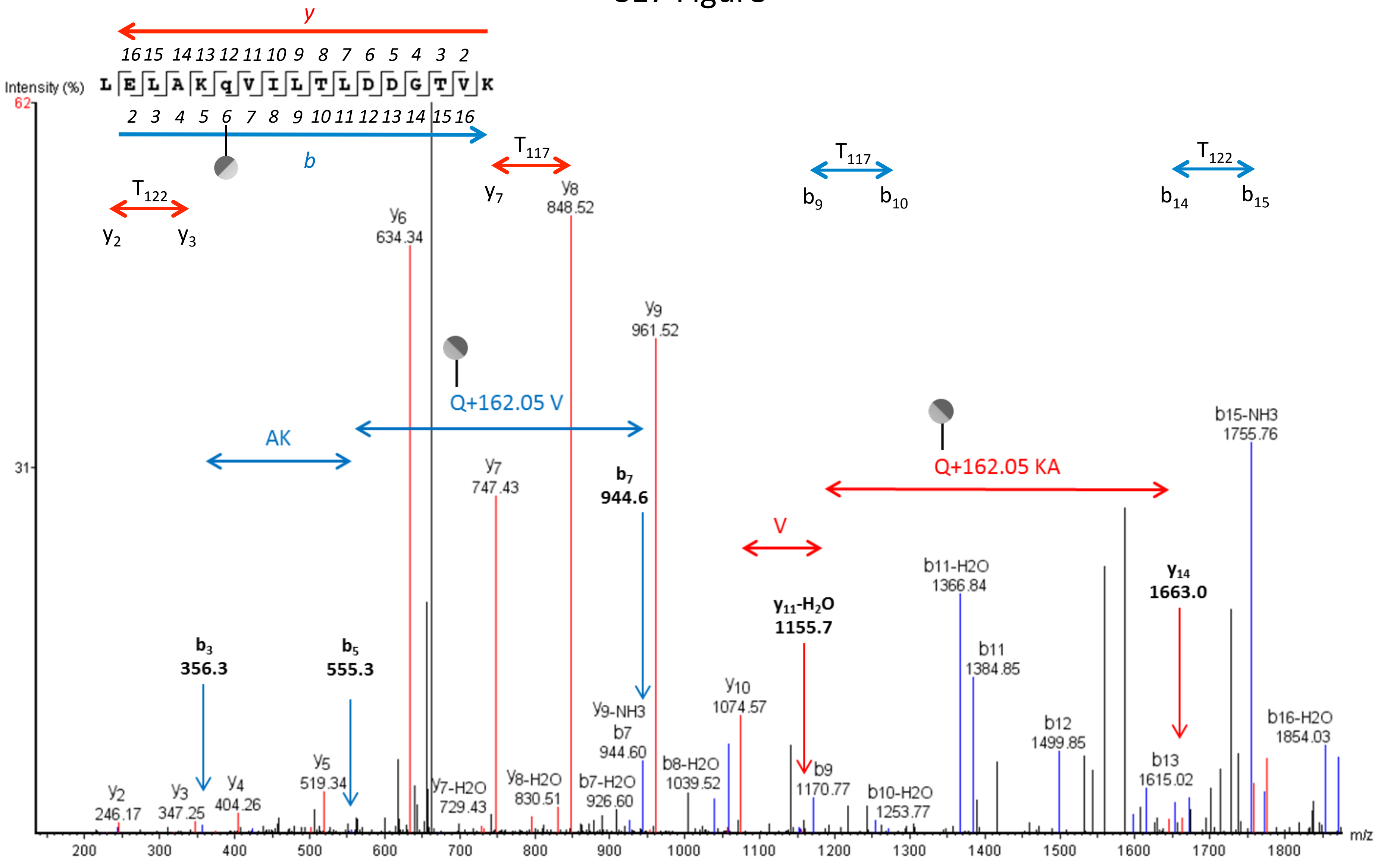

S17 Fig. LC MS/MS-CID showing hexosylation at Gln113 of the peptide LELAKq_{113}VILTLDDGTVK of MARTH_403. The assigned b and y ions are shown in blue and red, respectively. Glycosylation of T glycosites is absent in this spectrum as illustrated. The PEAKS peptide score (-10lgP) for this spectrum was 66. The charge state of the parental ion was $z = 3$.
