# Supplementary material for: General N-and O-Linked Glycosylation of Lipoproteins in Mycoplasmas and Role of Exogenous Oligosaccharide
Source: PLoS One. 2015 Nov 23;10(11):e0143362. doi: 10.1371/journal.pone.0143362 (PMC4657876; doi:10.1371/journal.pone.0143362)
Supplement: S1 Table — (PDF) [file pone.0143362.s018.pdf]

S1 Table. MS/MS peak assignments for the peptide Gt_{64}KDFLPIELQSLEVSK of MYPU_3230

m/z	assignment	*m/z*	assignment
321.2	b_2	216.1	y_2-H_2O
356.5	b_5[2+]	234.1	y_2
412.8	b_6[2+]	315.0	y_3-H_2O
432.3	b_3-NH_3	333.2	y_3
461.3	b_7[2+]	444.2	y_4-H_2O
711.3	b_5	462.3	y_4
806.3	b_6-H_2O	516.8	y_9[2+]
824.3	b_6	575.3	y_5
903.4	b_7-H_2O	644.3	y_6-H_2O
1016.4	b_8-H_2O	662.3	y_6
1034.4	b_8	678.3	y_{12}[2+]
1145.5	b_9-H_2O	773.4	y_7-NH_3
1163.5	b_9	790.4	y_7
1276.7	b_{10}	809.3	y_{14}[2+]
1386.5	b_{11}-H_2O	873.3	y_{15}[2+]
1404.5	b_{11}	903.4	y_8
1473.7	b_{12}-H_2O	1005.1	y_{16}[2+]
1491.5	b_{12}	1014.3	y_9-H_2O
1586.7	b_{13}-H_2O	1032.5	y_9
1604.7	b_{13}	1127.4	y_{10}-H_2O
1715.7	b_{14}-H_2O	1145.5	y_{10}
1733.7	b_{14}	1242.6	y_{11}
1814.8	b_{15}-H_2O		
1832.8	b_{15}		
1901.8	b_{16}-H_2O		
1919.7	b_{16}		
