# Supplementary material for: General N-and O-Linked Glycosylation of Lipoproteins in Mycoplasmas and Role of Exogenous Oligosaccharide
Source: PLoS One. 2015 Nov 23;10(11):e0143362. doi: 10.1371/journal.pone.0143362 (PMC4657876; doi:10.1371/journal.pone.0143362)
Supplement: S2 Table — (PDF) [file pone.0143362.s019.pdf]

S2 Table. MS/MS peak assignments for the peptide STLEYTINNSQELQn$_{335}$ILKQTYEEFTK of MYPU_3200

m/z	assignment	*m/z*	assignment
413.0	b_4-H_2O	377.1	y_3-H_2O
431.1	b_4	395.1	y_3
576.3	b_5-H_2O	506.3	y_4-H_2O
594.2	b_5	507.2	y_4-NH_3
626.7	b_{11}[2+]	524.2	y_4
677.3	b_6-H_2O	635.2	y_5-H_2O
695.3	b_6	643.4	y_{10}[2+]
790.3	b_7-H_2O	653.2	y_5
808.3	b_7	813.3	y_6
904.4	b_8-H_2O	902.4	y_{13}[2+]
922.3	b_8	917.3	y_7
949.6	b_{15}[2+]	1027.0	y_8-H_2O
1018.4	b_9-H_2O	1045.3	y_8
1019.5	b_9-NH_3	1173.3	y_9
1062.8	b_{17}[2+]	1269.4	y_{10}-NH_3
1106.3	b_{10}-NH_3	1286.7	y_{10}
1123.3	b_{10}	1675.5	y_{12}
1190.9	b_{19}[2+]	2242.8	y_{17}-H_2O
1243.4	b_{11}-NH_3	2374.8	y_{18}
1362.4	b_{12}-H_2O	2470.8	y_{19}-H_2O
1363.6	b_{12}-NH_3	2489.0	y_{19}
1380.5	b_{12}	2583.8	y_{20}-H_2O
1387.0	b_{22}[2+]	2602.0	y_{20}
1475.5	b_{13}-H_2O	2685.0	y_{21}-H_2O
1476.7	b_{13}-NH_3	2703.0	y_{21}
1493.6	b_{13}	2848.0	y_{22}-H_2O
1604.7	b_{14}-NH_3	2866.2	y_{22}
1621.4	b_{14}	2977.2	y_{23}-H_2O
2233.8	b_{18}-H_2O	2995.0	y_{23}
2251.7	b_{18}	3210.0	y_{25}
2462.8	b_{20}-H_2O		
2626.2	b_{21}-H_2O		
2644.0	b_{21}		
2754.8	b_{22}-H_2O		
2902.0	b_{23}		
3031.0	b_{24}-H_2O		
3132.0	b_{25}-H_2O		
