# Supplementary material for: General N-and O-Linked Glycosylation of Lipoproteins in Mycoplasmas and Role of Exogenous Oligosaccharide
Source: PLoS One. 2015 Nov 23;10(11):e0143362. doi: 10.1371/journal.pone.0143362 (PMC4657876; doi:10.1371/journal.pone.0143362)
Supplement: S3 Table — (PDF) [file pone.0143362.s020.pdf]

S3 Table. MS/MS peak assignments for the peptide ANAKn_{495}FYGFSDAYGK of MARTH_403

m/z	assignment	*m/z*	assignment
186.2	b_2	186.2	y_2-H_2O
257.2	b_3	204.1	y_2
367.3	b_4-H_2O	219.4	y_4[2+]
643.6	b_5-H_2O	349.8	y_3-NH_3
644.5	b_5-NH_3	367.3	y_3
661.3	b_5	394.3	y_7[2+]
790.4	b_6-H_2O	420.2	y_4-H_2O
808.4	b_6	438.3	y_4
953.4	b_7-H_2O	535.9	y_5-NH_3
971.5	b_7	553.3	y_5
1010.4	b_8-H_2O	640.3	y_6
1028.4	b_8	769.4	y_7-H_2O
1158.0	b_9-H_2O	787.4	y_7
1175.7	b_9	827.5	y_8-NH_3
1244.5	b_{10}-H_2O	844.4	y_8
1262.5	b_{10}	990.4	y_9-NH_3
1359.7	b_{11}-H_2O	1007.4	y_9
1360.6	b_{11}-NH_3	1154.3	y_{10}
1377.5	b_{11}	1412.5	y_{11}-H_2O
1430.7	b_{12}-H_2O	1430.7	y_{11}
1448.5	b_{12}	1541.7	y_{12}-NH_3
1593.7	b_{13}-H_2O	1558.7	y_{12}
1650.7	b_{14}-H_2O	1611.8	y_{13}-H_2O
1668.8	b_{14}	1629.7	y_{13}
		1743.7	y_{14}
