# Supplementary material for: General N-and O-Linked Glycosylation of Lipoproteins in Mycoplasmas and Role of Exogenous Oligosaccharide
Source: PLoS One. 2015 Nov 23;10(11):e0143362. doi: 10.1371/journal.pone.0143362 (PMC4657876; doi:10.1371/journal.pone.0143362)
Supplement: S4 Table — (PDF) [file pone.0143362.s021.pdf]

S4 Table. MS/MS peak assignments for the peptide ITDLLSq$_{49}$KEVTETQK of MYPU_3460

m/z	assignment	*m/z*	assignment
215.1	b_2	258.2	y_2-NH_3
312.1	b_3-H_2O	275.2	y_2
330.1	b_3	376.1	y_3
425.1	b_4-H_2O	487.4	y_4-H_2O
443.2	b_4	488.3	y_4-NH_3
467.1	b_7[2+]	505.3	y_4
539.2	b_5-NH_3	588.3	y_5-H_2O
556.3	b_5	589.2	y_5-NH_3
626.0	b_6-NH_3	606.3	y_5
810.8	b_{13}[2+]	705.3	y_6
915.4	b_7-H_2O	817.7	y_7-NH_3
1044.5	b_8-NH_3	834.3	y_7
1061.6	b_8	944.3	y_8-H_2O
1172.4	b_9-H_2O	962.3	y_8
1190.6	b_9	1321.3	y_{10}-H_2O
1271.5	b_{10}-H_2O	1339.5	y_{10}
1289.5	b_{10}	1434.5	y_{11}-H_2O
1372.5	b_{11}-H_2O	1452.5	y_{11}
1390.5	b_{11}	1547.5	y_{12}-H_2O
1519.3	b_{12}	1565.5	y_{12}
1603.5	b_{13}-NH_3	1662.6	y_{13}-H_2O
1730.5	b_{14}-H_2O	1680.5	y_{13}
1748.5	b_{14}	1763.5	y_{14}-H_2O
		1781.6	y_{14}
