# Supplementary material for: General N-and O-Linked Glycosylation of Lipoproteins in Mycoplasmas and Role of Exogenous Oligosaccharide
Source: PLoS One. 2015 Nov 23;10(11):e0143362. doi: 10.1371/journal.pone.0143362 (PMC4657876; doi:10.1371/journal.pone.0143362)
Supplement: S5 Table — (PDF) [file pone.0143362.s022.pdf]

S5 Table. MS/MS peak assignments for the peptide LELAKq_{113}VILTLDDGTVK of MARTH_40

m/z	assignment	*m/z*	assignment
225.3	b_2-H_2O	228.8	y_2-NH_3
243.2	b_2	246.2	y_2
356.3	b_3	318.1	y_6[2+]
423.3	b_6[2+]	347.3	y_3
555.3	b_6	374.2	y_7[2+]
926.6	b_7-H_2O	404.3	y_4
944.6	b_7	501.3	y_5-H_2O
1039.5	b_8-H_2O	519.3	y_5
1057.7	b_8	634.3	y_6
1152.7	b_9-H_2O	729.4	y_7-H_2O
1153.6	b_9-NH_3	732.4	y_{12}[2+]
1170.8	b_9	796.4	y_{13}[2+]
1253.8	b_{10}-H_2O	830.5	y_8-H_2O
1271.8	b_{10}	848.5	y_8
1366.8	b_{11}-H_2O	944.6	y_9-NH_3
1384.8	b_{11}	953.5	y_{16}[2+]
1499.9	b_{12}	961.5	y_9
1597.0	b_{13}-H_2O	1056.4	y_{10}-H_2O
1615.0	b_{13}	1057.7	y_{10}-NH_3
1653.9	b_{14}-H_2O	1074.6	y_{10}
1672.0	b_{14}	1155.7	y_{11}-H_2O
1755.8	b_{15}-NH_3	1644.8	y_{14}-H_2O
1773.2	b_{15}	1663.0	y_{14}
1854.0	b_{16}-H_2O	1758.0	y_{15}-H_2O
1872.0	b_{16}	1776.0	y_{15}
